# STELLAR: A Fragment-Based Docking Workflow for Highly Flexible Long-Chain Biopolymers

**DOI:** 10.1021/acs.jcim.6c00987

**Published:** 2026-09-16

**Authors:** Alejandro Rodríguez-Martínez, Jochem Nelen, Miguel Carmena-Bargueño, Carlos Martínez-Cortés, Horacio Pérez-Sánchez

**Affiliations:** Structural Bioinformatics and High Performance Computing Research Group (BIO-HPC), 16728 UCAM Universidad Católica de Murcia (UCAM), 30107 Murcia, Spain

## Abstract

Docking methods have improved significantly through optimization using generative approaches and systematic exploration of parameter configurations within docking software. However, docking large molecules such as peptides remains challenging, while the docking of large non-peptidic biopolymers is still insufficiently explored. Existing tools often exhibit limitations in both accuracy and computational efficiency when applied to longer peptides or other polymers. Moreover, although generative deep learning and machine learning approaches have shown promise, they still frequently lack robust ranking metrics, resulting in suboptimal performance. To address these limitations, we developed STELLAR (Score-Tuning for Efficient Ranking of Large Ligands using an Accurate and Refined Docking Configuration), a workflow designed to enable efficient and accurate docking of highly flexible long-chain biopolymers exceeding 10 subunits. STELLAR implements a fragment-based strategy, decomposing the biopolymer into smaller units for individual docking, followed by recomposition into full-length structures. It also includes optimized algorithms for handling long-chain biopolymers. The pipeline integrates structural optimization steps using tools such as GNINA, RDKit, and GROMACS, ensuring physically realistic poses. STELLAR achieved RMSD values below 5 Å in validation experiments using benchmark complexes from Propedia and the Protein Data Bank (PDB). It offers a faster and more efficient alternative to several state-of-the-art tools across a range of peptide lengths. Additionally, it scales linearly in computational time, runs primarily on CPUs with minimal GPU usage, avoids reliance on generative approximations, provides an accurate metric for ligand ranking, and reduces computational cost compared to similar tools. Its design also supports high-throughput screening of linear polymer–protein interactions by efficiently reducing the complexity associated with high conformational flexibility.

## Graphical abstract

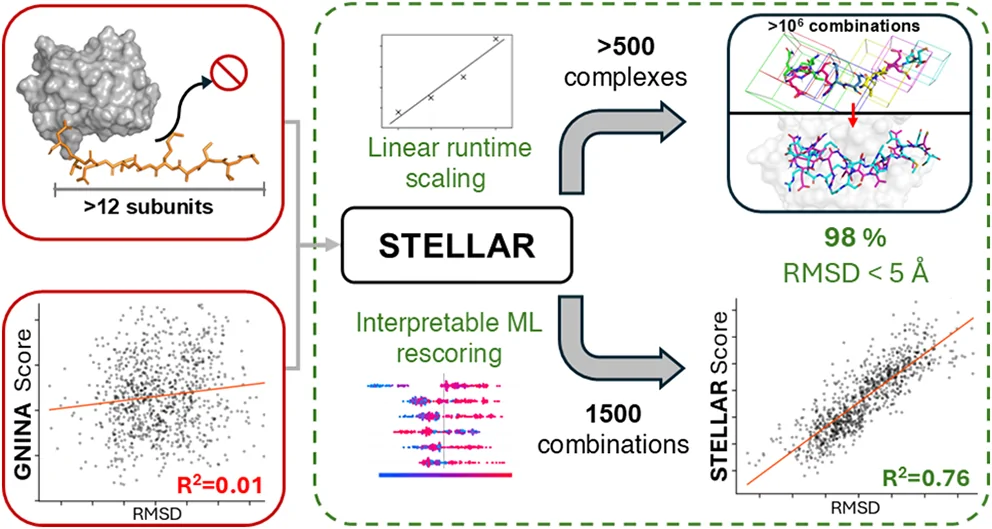

## Introduction

Developing new drugs is a lengthy and costly process, with expenses ranging from hundreds of millions to over 4 billion USD, largely due to preclinical and clinical testing requirements.^1^ Hit discovery, often initiated through high-throughput screening (HTS), adds to these costs but is limited by the vastness of chemical space. Virtual screening (VS) offers a faster, more cost-effective alternative by computationally narrowing down candidate compounds, thus reducing the need for experimental testing.^2^

Among the VS techniques, molecular docking is widely used. It requires 3D structures of the target and ligand to predict optimal binding poses and score their affinity, helping to prioritize promising leads for further study. Docking-based virtual screening represents a cornerstone of structure-based drug design and is extensively employed by the scientific community to facilitate the identification of novel lead compounds targeting therapeutically relevant biomolecules.^3^

Although ligand–protein docking has been an active area of research in recent years, significant challenges remain when dealing with long-chain polymers, for which current methodologies often show reduced accuracy and efficiency.

In the complex landscape of molecular interactions, there is a vast combinatorial space for all of the possible molecular structures. Atomic-resolution structures of protein–ligand complexes are fundamental in drug design. The multitude of variations in molecular structures leads to an equally vast number of possible complexes. This structural diversity, coupled with the inherent complexity of the interaction between proteins and long-chain polymers, has fostered the evolution of diverse experimental and computational methodologies. However, determining the large-scale structure of such complexes poses a significant challenge, even for high-throughput screening methods.^4,5^

Given the need to overcome the limitations of traditional experimental methods, theoretical approaches can serve as a promising alternative. Molecular docking of the peptide and other linear polymers to their target proteins presents a viable theoretical approach. However, this approach has practical challenges. This kind of polymer chain might be too large for classical or fast docking approaches,^6^ since its extensive flexibility results in a complex search problem.^7,8^ Moreover, in many cases, even the approximate location of the peptide binding sites on the target surface is unknown.^8^ To try to solve this problem, several approaches have been developed that scan the entire protein surface to detect potential protein–ligand interaction zones. However, modeling the docking of long-chain polymers to proteins presents significant challenges.

From a general perspective, we can highlight that some of the key challenges include managing the flexibility and complexity of these macromolecular structures, the computational cost, and the lack of suitable methods for efficiently simulating the interactions between proteins and long-chain polymers. These challenges are discussed below in the following sections, where we have conducted an exhaustive state-of-the-art search taking into account various variations and combinations of the keywords molecular docking, protein–peptide, protein–polymer, flexibility, benchmark, molecular dynamics, coarse-grained, high-performance computing, among others.

The computational implementation of flexibility in peptide–protein interactions presents several challenges. A crucial aspect is the characterization of the affinity and binding mechanism of specific molecules to a protein’s active site, which is relevant both scientifically and industrially. In principle, this information can be obtained through molecular dynamics (MD) simulations, although they are a computationally demanding task. Coarse-grained (CG) models are currently being applied to the study of large biomolecular systems or those that require long simulation times. For example, protein flexibility, induced by different elastic network models compatible with the Martini force field, has been tested in the calculation of free-energy profiles for protein–ligand binding, reflecting the importance of including greater flexibility in the parametrization of protein CG. Flexibility is also evident in myelin basic protein (MBP), which is intrinsically disordered and changes its structure and dynamics in response to a denaturant, demonstrating a relationship between flexibility and the structural expansion caused by the denaturant.^9^

These investigations show that although considerable progress has been made, conformational flexibility and intrinsic disorder in proteins continue to present significant challenges for structural biology and drug design. Flexibility and disorder can dramatically affect membrane stability, interactions with ligands and other proteins, and biological functions. Therefore, an accurate representation and understanding of flexibility and disorder in proteins are crucial to advancing these fields.

At present, even the most pioneering tools in the docking and molecular structure prediction field, such as AlphaFold3,^10^ Boltz,^11^ and Chai,^12^ are not specifically designed to perform virtual screening of large-chain peptides or other large flexible ligands while providing a dedicated scoring function for systematically ranking the predicted binding poses. In addition, these tools are mainly focused on protein–peptide systems, and their applicability to other classes of biopolymers remains limited. STELLAR (Score-Tuning for Efficient Ranking of Large Ligands using an Accurate and Refined docking configuration) addresses this limitation through a workflow that can be applied to different types of biopolymers and that provides affinity-related metrics for pose and ligand ranking.

## State of the Art

The implementation of modeling methods for protein–ligand interactions faces significant computational obstacles. Detailed atomic-level simulations, although accurate, are computationally demanding and limit their large-scale application.^13^ Molecular docking is computationally expensive, especially when analyzing large databases of protein structures, although combining ligand- and receptor-structure-based approaches can reduce runtimes.^14,15^ Hybrid approaches combining molecular mechanics with coarse-grained or Monte Carlo methods have been presented to predict ligand poses with lower computational costs.^16,17^ The use of supercomputers and machine learning techniques can help streamline the drug discovery process.^18,19^ Long time-step protocols can delay simulations of the protein–ligand recognition process rather than accelerate them. Efficient estimation of binding energy and binding kinetics is crucial for ligand optimization, yet it remains a considerable computational challenge.^20,21^ These obstacles underscore the need to develop more efficient and accurate methods to manage the complexity and computational cost associated with modeling protein–ligand interactions.

The current landscape lacks computational methods to efficiently simulate interactions between proteins and long-chain polymers such as peptides. This technological gap represents a significant obstacle to the discovery of bioactive compounds derived from long-chain polymers, resulting in a missed opportunity. Examples of these polymeric structures include polypeptide chains with more than five units, nucleic acids, polysaccharides, and so on. Conventional simulation methods fall short in their ability to perform these calculations, thus inhibiting progress toward the discovery of new drugs based on these polymeric structures.

Liu et al.^22^ presented the CB-Dock2 server for protein–ligand blind docking, reporting an 85% success rate in pose prediction; however, it does not efficiently handle long polymers. Che et al.^23^ developed a blind docking strategy for binding site prediction, although it is restricted to small ligands. The DSDP^24^ leverages machine learning to accelerate docking but does not adequately capture the complexity of polymeric ligands. Similarly, Huang et al.^24^ introduced a GPU-accelerated version of DSDP, which remains unsuitable for polymers. Grasso et al.^25^ proposed FRAD to explore protein volume, although its applicability to long polymers is limited.

Other methods, such as PatchMAN,^26^ ATPdock,^27^ and Attracting Cavities 2.0,^28^ are designed for specific applications and are not directly applicable to general polymer docking. Likewise, the approaches by Shanker and Sanner^29^ focus on protein–peptide interactions without extending to longer polymers. METADOCK2 ^30^ also improves computational speed and performance but remains limited to short-chain polymers. Overall, current methods do not scale efficiently to long polymers and fail to adequately capture their conformational complexity.

Recent reviews have highlighted the persistent lack of viable molecular docking solutions for long polymers. Although several approaches have been proposed, including fragment-based methods and general-purpose docking platforms, these strategies remain limited in their scope. Fragmentation approaches have been successfully applied to short peptides but do not scale efficiently to larger polymeric systems, while widely used docking software has not been specifically designed to address the challenges posed by long polymers.^31–35^

Rester^36^ proposed a fragmentation protocol, demonstrating difficulties with long polymers. Neudert and Klebe^37^ developed DSX to accelerate docking, but it was not designed for polymer complexity. The work of Liu and Cao^38^ describes the CB-Dock2 server for protein–ligand blind docking, without focusing on polymers.

Che et al.^23^ proposed improved blind docking to predict binding sites but were limited to small ligands. The method of Huang et al.^24^ accelerates blind docking with GPUs but does not address polymers. Grasso et al.^25^ extensively explored protein volume with FRAD with restrictions on long polymers. Finally, HPepDock,^39^ DockThor,^40^ and DLPepDock^41^ are currently the most efficient methods for peptide–protein docking, but they are limited to 12 residues, in the cases where it has been tested.

AI assistance techniques have also been tried in this challenge. Jin et al.^42^ and Zhang et al.^43^ try to use deep learning models to improve the peptide–protein docking via TPepDock and DistPepFold, respectively. However, although they can process larger peptides than methods without AI assistance, they lack the capability to offer a robust scoring function and to provide an accurate ranking for appropriately sorting the ligands.

Boltz-2 ^44^ and AlphaFold2 ^45^ have demonstrated important progress in the protein–peptide prediction using AI assistance to improve docking efficiency and precision but still are not adequate to modulate other kinds of large polymers such as polysaccharides.

Specialized carbohydrate-docking approaches such as VinaCarb,^46^ GlycoTorch Vina,^47^ and more recently water-guided docking strategies have improved the treatment of glycosidic conformations and protein–glycan interactions.^48^ However, these methods still face difficulties when dealing with large and highly flexible polymers, where the rapidly increasing conformational space complicates both pose generation and ranking. Consequently, the development of efficient strategies for exploring long-chain biopolymers while providing robust ranking metrics remains an active area of research.

After analyzing the state of the art, the following technological limitations were identified that hinder progress in the discovery of new polymer-derived bioactive compounds and can be resolved by implementing the presented planning.

1. Lack of effective methodologies: Although several approaches have been proposed, important limitations remain when modeling medium-to-large-sized polymeric ligands. Challenges related to conformational complexity, together with the lack of robust pose ranking strategies, continue to limit the applicability of current methodologies. This lack of adequate tools impedes the detailed exploration of the molecular interactions involved.
2. Chain size limitations: Existing methodologies are restricted in terms of the chain sizes they can process. In the best-case scenarios, they can handle only polypeptide chains of up to 5 units, which is insufficient to represent and study long-chain polymers that could have significant biological and pharmacological relevance.
3. Impact on the discovery of bioactive compounds: These technological limitations lead to a drastic restriction in the discovery process of polymer-derived bioactive compounds. The inability to simulate interactions between proteins and long-chain polymers limits the identification and validation of new bioactive compounds that could play crucial roles in various pharmacological and nutraceutical applications.

These limitations underscore the pressing need to develop new methodologies that can address these technological gaps and enable significant progress in the field of bioactive-compound discovery. The proposed project focuses on overcoming these technological barriers by developing an innovative fragment-based docking (FBD) methodology that aims to facilitate the efficient and accurate simulation of interactions between proteins and long-chain polymers.

Considering these limitations, the main objective is to take a step further toward the discovery of new knowledge and greater understanding in the scientific and technological field of computational chemistry by developing and validating computational models that allow for highly accurate predictions of the efficacy and safety of new bioactive compounds in the large ligand context as well as proposing promising candidates for experimental validation in future research stages.

The primary aim of this work was to develop new approaches for docking and ranking polymers that are not feasible to process using standard docking methods. We initially focused on peptide–protein docking due to the availability of extensive benchmarks and the fact that these systems are well studied. This made them suitable for evaluating the ability of our pipeline to reproduce known complexes involving linear polymers with a high number of rotatable bonds.

Although STELLAR was extensively tested on a large number of protein–peptide complexes, we also aimed to assess its performance on other types of biopolymer–protein interactions. To this end, we included polysaccharide–protein complexes, which are significantly less explored than peptide systems but are of considerable importance for the identification of novel hits in diverse biological contexts.

Although a direct quantitative comparison is difficult due to differences in benchmark composition, peptide length, and evaluation criteria, the performance of STELLAR is within the range reported for current state-of-the-art peptide-docking methods. CABS-dock reported that more than 80% of benchmark cases yielded medium- or high-quality models using a peptide RMSD threshold of 5.5 Å, whereas DockThor achieved a 40% success rate using a more stringent backbone RMSD threshold of 2.5 Å on a benchmark of 53 protein–peptide complexes containing peptides of up to 12 residues.^22,42^ Similarly, HPepDock reported a success rate of 33.3% in blind peptide docking on a benchmark of 57 complexes.^37^

Most of these benchmarks focus on relatively short peptides, reflecting the well-known challenge that docking accuracy decreases as peptide length and conformational flexibility increase due to the larger number of rotatable bonds and accessible conformations. In contrast, STELLAR was evaluated on a larger benchmark comprising 257 protein–peptide complexes and achieved RMSD values below 5 Å while handling longer and highly flexible peptide systems. Therefore, although these results should be interpreted cautiously due to differences in benchmark design, they suggest that STELLAR provides competitive structural predictions while extending the applicability of docking workflows to more challenging peptide systems. A rigorous head-to-head comparison against existing tools on a common benchmark would be valuable future work.

In addition, we aimed to develop a tool that achieves a balance between computational efficiency and the ability to produce physically realistic results while also enabling the ranking of different ligands using reliable docking metrics. Many existing methods capable of predicting poses for large peptides or other ligands rely heavily on the GPU resources. In contrast, although our workflow incorporates molecular dynamics simulations, we have sought to minimize GPU usage across the pipeline as much as possible.

In this way, STELLAR improves computational efficiency by parallelizing the docking calculations for each polymer fragment and distributing the docking process across independent grids, enabling the simultaneous exploration of the selected search region. The overall computational cost is dominated by the fragment docking calculations and therefore scales approximately linearly with the number of polymer fragments, while the remaining stages of the workflow have a negligible impact on the execution time.

Table 1 compares STELLAR to other docking software packages and highlights its computational efficiency and competitive execution times for docking simulations.

**Table 1:** Different Structure-Based Virtual Screening Tools Compared Some of Their Features; SD: Standard Docking, BD: Blind Docking

software	ligand input	max rotatable bonds tested	rank score	generative method	CPU estimated time^a^	GPU usage (%)^b^
AutoDock Vina	Small molecules	20–25	AutoDock Vina score	No	Not feasible	No
CB–Dock2	Small molecules	20–25	AutoDock Vina score	No	Not feasible	No
METADOCK2	Small molecules	20–25	METADOCK score	No	Not feasible	80
Boltz-2	Small molecules^c^	20–25	Affinity value	Yes	Not feasible	80–95
CABS-dock	Peptides	50–60	Not reported	No	180	No
HPepDock	Peptides	50–60	Custom docking score	No	125	No
DockThor	Peptides and other kinds of ligands	40–50	Total energy	No	240–360	No
DLPepDock	Peptides	80–100	Not reported	No	100–120	No
InterPepScore	Peptides	50–60	DockQ proxy	GNN	Not reported	No
TPepPro	Peptides	100–120	Not reported	GNN	Not reported	80–95
DistPepFold	Peptides	290–300	Not reported	AFM	10–30	80–95
**STELLAR**	**Any linear polymers (peptides, polysaccharides)**	**100–120**	**Gnina, STELLAR score, Total energy**	**No**	**120** ^d^	**10** ^e^

a Approximated values.

b Trained with small molecules (>50 heavy atoms).

c Time calculated in **minutes** for a standard docking of the largest molecule tested for the method.

d Running for a 1 single combination for 7 fragments peptide.

e GPU limited to optional GROMACS relaxation.

## Methods

STELLAR offers a massive exploration of the complete protein surface using various kinds of tools and an exhaustive exploration of the program’s configurable parameters to obtain poses with the greatest variability for each fragment.

The STELLAR workflow is primarily divided into three steps:

1. Fragmentation of the original peptide and generation of the polymer subunit libraries for the specific fragments (di- and tripeptides, di- and trisaccharides).
2. Docking calculations were performed for each of the fragments from the original peptide using GNINA docking software with a combination of the different chosen parameters to explore the docking space deeply and obtain a conformation pool for each fragment.
3. Filtering and joining of combinations of the fragments to generate the final poses of the complete polymer were done through an algorithm designed to obtain valid combinations and post-processing through a very short molecular dynamics program using GROMACS to relax the peptide structure and obtain the final analysis results.

The workflow generates different metrics that are processed and collected in a postanalysis step after Step 3, producing a final dataset containing all of the metrics for each valid combination of already reassembled fragments. Figure S1 illustrates the overall process, combining MetaScreener, STELLAR, GROMACS, and Sibila to provide a general overview and a more exhaustive analysis of the polymer docking results obtained from this workflow.

In the first place, we run the whole process for the protein–peptide complex, as the first test to validate STELLAR. Finally, we extended the workflow to another polymer family, polysaccharides, to demonstrate the polivalence of the tool in different contexts of polymers. For that, a benchmark from a subset of complexes generated from protein–peptide with the PDB crystallographic associated database Propedia^49^ was used as the principal benchmark to test STELLAR. Also, after testing the protein–peptide cases, we chose eight carbohydrate–protein complexes to check the versatility of STELLAR.

### Benchmark Design

In order to validate the accuracy of STELLAR for docking large linear polymers, benchmark datasets consisting of protein–peptide and carbohydrate–peptide complexes were developed to evaluate the performance of STELLAR in realistic cases.

Protein–peptide complexes were extracted from the Propedia database due to the availability of crystallographic structures in the Protein Data Bank (PDB), which enabled the calculation of RMSD values and other validation metrics for the tool. Complexes were retrieved from the Propedia webpage (https://bioinfo.dcc.ufmg.br/propedia/, accessed November 18, 2025) to compare the RMSD values with the final simulated poses obtained through the STELLAR workflow. The corresponding receptor structures were downloaded and preprocessed using the AutoDockTools automatic pipeline (using the pdbqtconvert.sh shell script) in order to perform docking with GNINA.

For each complex, the peptide ligand was also extracted separately to allow a comparison between the docked or simulated poses and the fragments derived from the crystallographic peptide. The center of mass of each crystallographic fragment was calculated and assigned as the reference coordinate for the fragment. These coordinates were subsequently used to generate the corresponding commands required to launch MetaScreener for the direct GNINA docking. All generated files were organized into separate folders to ensure proper organization.

In addition, protein–carbohydrate complexes involving linear polysaccharide ligands were selected based on chain length and monosaccharide composition. For this benchmark, eight PDB entries containing proteins complexed with linear polysaccharides composed of 8–10 monosaccharide units were selected. These cases were used to evaluate the applicability of STELLAR to other types of linear polymers beyond peptides, requiring only minor modifications to the code to enable the processing of carbohydrate ligands.

To identify suitable systems, a script was developed using the AI-assisted programming tool Cursor, which enabled interaction with the PDB-MCP Server (https://github.com/Augmented-Nature/PDB-MCP-Server). This tool was used to search for crystallographic PDB complexes containing linear polysaccharides, using ligand type, chain length, and monosaccharide composition as selection criteria. Eight representative protein–polysaccharide complexes were selected, covering ligands with lengths ranging from 6 to 10 monosaccharide units and diverse chemical compositions.

### Fragmentation and Library Generation

STELLAR was designed to efficiently handle peptide docking for a wide range of chain lengths, particularly medium- to long-peptides. Later, we tested the protocol for a different kind of biomacromolecule, like polysaccharides, to check the polivalence of our tool.

The first step in the workflow involves fragmenting the original peptide into smaller units that can be independently docked. To achieve this, several molecular modeling tools were integrated, including PyMOL,^50^ Maestro (Schrödinger),^51^ RDKit.^52^ Each tool was selected for its specific advantages in molecular fragmentation, atom-level manipulation, or structural conversion, depending on the context.

A central component of the methodology is the creation of a comprehensive fragment library. For peptide–protein cases, 400 dipeptides and 8000 tripeptides were generated to encompass all possible combinations of standard amino acids. This extensive library serves as a foundation for reconstructing longer peptides from smaller chemically relevant building blocks, providing broad coverage of structural diversity and ensuring adaptability across peptide targets. This library includes only combinations of the 20 standard amino acids; however, users may extend the fragment database with custom dipeptides and tripeptides containing non-canonical residues or post-translational modifications. A reduced-carbohydrate-fragment-based library was generated with the subunits present in the PDB cases analyzed for this study.

Fragment generation was initially conducted in PDB format without 3D coordinates using PyMOL scripting. These initial representations were then processed and converted using in-house scripts for compatibility with subsequent docking workflows. First, RDKit and Maestro (Schrödinger) were used to modify the initial representations and generate three different kinds of fragments depending on the position in the final chain (start-end, internal end-chain fragments). The systematic structure preparation ensured that all fragments were uniformly formatted and ready for deployment across various docking engines. To achieve this, each fragment was prepared and exported in PDBQT formats using the Molconvert tool from AutoDockTools,^53^ following the same protocol for atom formatting and charge assignment across all fragments.

In the case of polysaccharide testing, the corresponding di- and trisaccharides were generated via CHARMM-GUI to generate the conformations. Subsequently, RDKit was used to delete and aggregate the atoms corresponding to the fragments, and the Molconvert protocol from AutoDockTools was used to export to PDBQT format.

The entire fragmentation and preparation process was developed with automation and scalability in mind. All steps were embedded in a modular scripting environment, enabling the batch processing of hundreds of peptides across high-performance computing (HPC) infrastructures. This design ensures reproducibility and enables the STELLAR workflow to handle large-scale virtual screening campaigns.

To validate the robustness of the fragmentation strategy, cross-platform testing was conducted across large peptides of varying lengths (from 12 to 21 residues). A carbohydrate approach was also tested for a nonasaccharide, generating the corresponding di- and trisaccharide library. The consistent performance across systems confirmed the reliability of the fragment generation protocols under the realistic molecular docking conditions.

This library generation pipeline was developed to enhance the versatility of STELLAR in the future, extending its applicability to other polymer types beyond peptides, including polysaccharides, fatty acids, and nucleic acids.

Finally, all generated fragments were processed for use within the MetaScreener platform (https://github.com/bio-hpc/metascreener), which manages input formatting, batch docking submission, and downstream analysis. By ensuring tight integration with this pipeline, the STELLAR framework supports the seamless docking of fragment libraries with minimal manual intervention, significantly accelerating computational workflows.

### Fragment Docking

Gnina^54^ docking software was selected as the docking engine within the STELLAR workflow due to its versatility, reliability, and computational efficiency, even when using elevated exhaustiveness parameters. An intermediate exhaustiveness value of 64 (default: 8) was employed to enhance the exploration of potential binding poses while maintaining a reasonable execution time. Additionally, an RMSD threshold of 2 Å (instead of the default 1 Å) was applied during pose filtering to reduce redundancy among the final docking conformations. The maximum number of binding modes was increased to 200. All docking tasks were orchestrated using the in-house high-throughput virtual screening framework MetaScreener, which integrates multiple docking engines into a unified and parallelized computational pipeline.

The docking process requires the definition of a grid box. For each fragment, its volume was calculated to define an appropriate grid size along the three spatial dimensions, while the grid center was determined on the basis of the original position of each region within the polymer. Each docking instance was executed as an independent job, enabling efficient large-scale parallelization.

As shown in Figure 1, the workflow begins with a shell script (ms.sh, located in the MetaScreener directory) that processes each peptide fragment and the corresponding protein target. These inputs are converted into a standardized format during the preparation stage, which includes file conversion, energy grid calculation, and job queue configuration. Each docking task (job 1 to job N) is then executed using one of the selected engines with its corresponding configuration.

**Figure 1:**
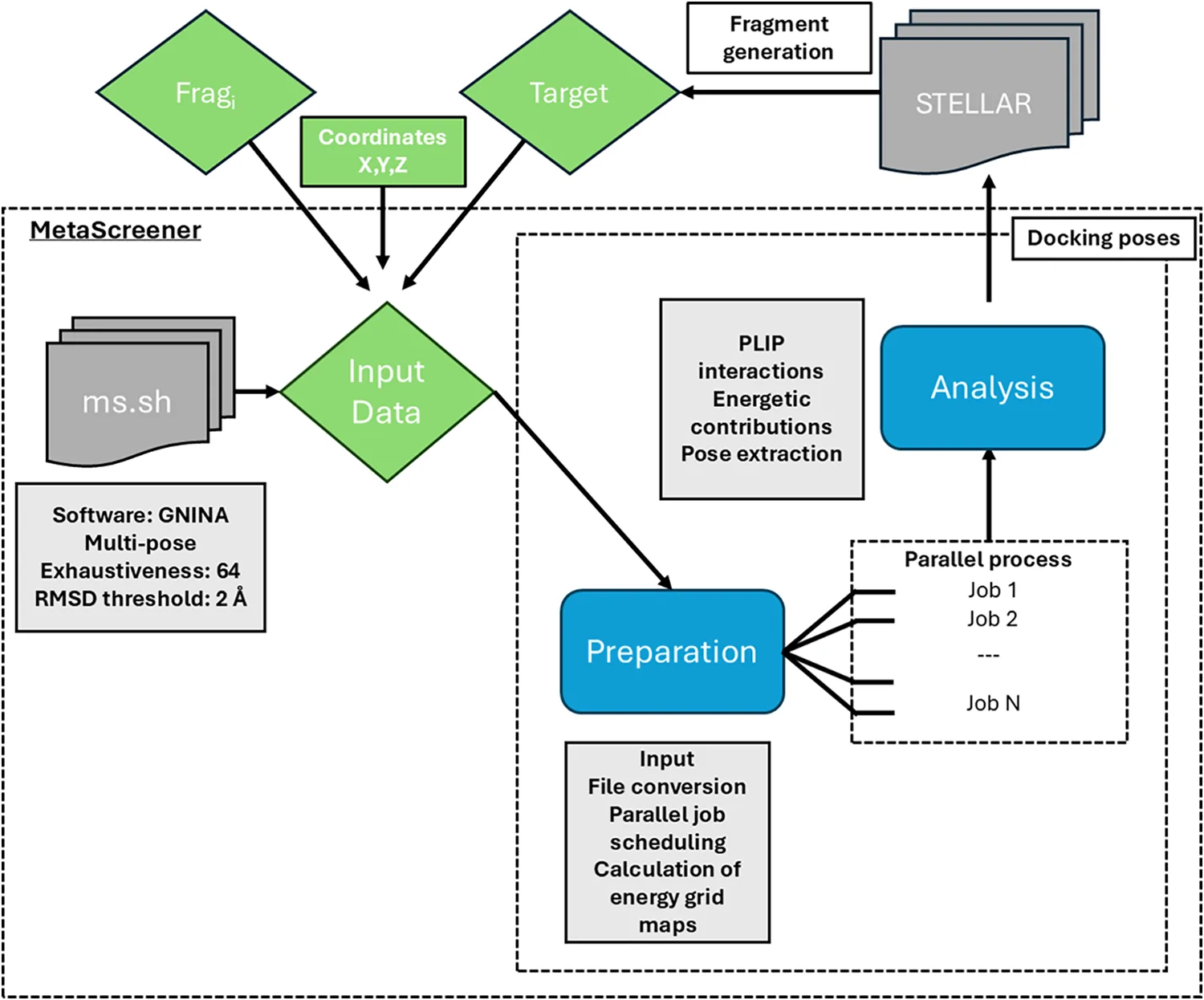
Schematic representation of the docking workflow for individual peptide fragments using the MetaScreener platform. The process begins with the execution of the ms.sh launcher script, which initializes input parameters, including the docking protocol, selected engine, and optional settings. These inputs are processed by STELLAR to fragment the full peptide sequence and define the coordinates for each fragment. Input files are parsed and standardized during the Input Data stage. The Preprocess module handles file conversion, job scheduling, and the calculation of energy grid maps for engines that require them. Docking jobs (Job 1 to Job N) are then distributed in parallel to maximize computational throughput. Upon completion, results are aggregated and processed in the Analysis stage, which includes visualization with PyMOL, interaction profiling using PLIP, and extraction of energetic contributions. The resulting data for each docking pose are then used by STELLAR in subsequent steps of the workflow.

Once the docking simulations were completed, the results were collected and processed during the analysis stage. At this step, interaction data are extracted, including pose-specific information and interaction diagrams generated using PLIP.^55^ All results can also be visualized through PSE files generated with PyMOL.^50^ The script get_histogram_picture.py is used to generate graphical summaries of docking score distributions, facilitating the pose prioritization and selection. Final outputs are stored in a structured format, including JSON files, docking scores, and 3D molecular structures.

This highly automated and modular docking protocol enables STELLAR to efficiently process peptide fragments across multiple docking engines, achieving a balance between the computational speed and accuracy. The output of this stage serves as an input for the subsequent phase of the workflow: fragment recombination and structural refinement.

### Filtering and Joining

Once all docking poses for each peptide fragment have been generated, the next step in the STELLAR workflow is to systematically filter and recombine these poses into full-length peptide conformations. Each fragment (e.g., di- or tripeptide, di- or trisaccharide) may yield multiple docking poses, generating a combinatorial explosion of potential assemblies across all fragments. To address this issue, STELLAR employs a set of custom scripts that reduces the search space by retaining only structurally viable and chemically coherent combinations.

Two key filters are applied: (1) peptide bond distance verification to maintain proper geometry between adjacent fragments and (2) steric overlap consistency between atoms of connecting fragments. Only combinations that satisfy these criteria are propagated to the subsequent steps.

Before executing the STELLAR scripts, the ms.sh script extracts the coordinates of the atoms involved in the repetitive bond of the corresponding polymer (C–N for peptide bonds, C1–O4 for glycosidic bonds 1 → 4, and C1–O3 for glycosidic bonds 1 → 3). This information is stored together with the docking results obtained in the previous stage.

STELLAR evaluates all possible pose combinations by computing the distance between the atoms involved in the critical bond connecting adjacent fragments. The default maximum distance threshold is 1.8 Å, although this value can be adjusted depending on the bond type or the atoms involved. Distances are calculated using the Euclidean distance between atomic coordinates. For protein–peptide complexes, the distance between the carbon and nitrogen atoms forming the peptide bond between consecutive fragment poses is calculated, considering the corresponding nitrogen atom of the first residue of the fragment pose and the corresponding carbon atom of the last residue of the previous fragment pose.

Combinations that satisfy the bond-distance criterion are subsequently evaluated using a second filter based on steric overlap between consecutive fragment poses in order to avoid clashes and unrealistic contacts. The overlap is calculated using

1 $${overlap}_{PoseF_{i}-PoseF_{i+1}}=\left({vol}_{PoseF_{i}}+{vol}_{PoseF_{i+1}}\right)-vol_{PoseF_{i}Pose\mathrm{Fr}ag_{i+1}}$$

In this step, a software tool based on the ideas of the software MURCIA^56^ is used to calculate the molecular volumes. Three different volumes are computed: the volume of the first fragment pose (vol_PoseF*_i_*_), the volume of the adjacent fragment pose (vol_PoseF_*i*+1__), and the combined volume occupied by both fragments (vol_PoseF*_i_* PoseFrag_*i*+1__) (eq 1). The steric overlap is then obtained as the difference between the sum of the individual volumes and the combined volumes of both fragments.

An overlap threshold of 350 Å^3^ is applied by default to discard invalid combinations. Similar to the distance criterion, this value can be adjusted depending on the characteristics of the studied system.

Starting from the poses of the first fragment and its adjacent fragment, an overlap filter is applied sequentially. Only those poses that satisfy the overlap criterion are propagated to the next fragment pair. In this way, the overlap check proceeds iteratively across the consecutive fragment pairs. As soon as one pair exceeds the overlap tolerance, the entire combination is rejected, and no further evaluations are performed.

To ensure that STELLAR could also be applied to carbohydrate polymers and other linear polymers, minimal modifications were introduced through the creation of a new code fork. These changes mainly affected the coordinate extraction and file manipulation scripts. Additional scripts were developed to correctly remap the atoms involved in each of the specific cases.

For carbohydrate systems, the distance filter considered the distance between the C4 atom of the last subunit of fragment 1 and the O4 atom of the first subunit of the adjacent fragment, as well as the C1 atom of the first subunit of the adjacent fragment. For glycosidic bonds of type 1 → 3, the O4 atom was replaced by the O3 atom of the first subunit of the adjacent fragment. A renaming step was applied to ensure compatibility with the distance-filtering script. The remainder of the workflow was executed following the standard protocol, producing the final results for each valid fragment combination in the corresponding CSV file together with their associated output files.

As illustrated in Figure S2, the total number of pose combinations across fragments (*X* × *Y* × *Z*, etc.) is drastically reduced during the filtering stage, and only the valid subsets are assembled into complete peptide structures. This strategy ensures both structural consistency and computational efficiency, providing a reliable basis for high-accuracy scoring and dynamic refinement in subsequent stages. Figure S3 shows the algorithm implemented in STELLAR to properly filter valid fragment combinations based on distance and overlap criteria.

### Recomposition

After the filtering steps, we obtained a specific number of valid pose combinations. STELLAR then processes these valid pose sets and geometrically joins them using their corresponding C- and N-terminal atoms to assemble full peptide chains. The resulting recombined structures represent realistic peptide candidates that maintain the correct connectivity and spatial conformation. This step is key for assembling biologically relevant peptide poses before the final structural refinement.

Finally, each of the validated pose combinations was joined using the RDKit package while keeping the initial geometry. A subsequent minimization is carried out to ensure the correct fit between the fragments that form the final polymer pose. For this purpose, different tools using the RDKit package were applied. Long bonds are shortened, pairs of atoms that are too close are relaxed to increase the distance between them and avoid possible clashes, and a minimization using MMFF is applied to the final assembled peptide to further reduce the number of clash problems. Finally, if there are charge problems in some atoms, then they are detected and adjusted to be valid for the next steps. Each of these final poses treated with RDKit is used to generate the topology and run a very short MD simulation to relax the complex before calculating the postanalysis metrics.

For each combination, a final assembly of the polymer is obtained. Each assembled polymer using the RDKIT package is driven toward short simulations via GROMACS, relaxing the structure. This process allows the removal of steric clashes and stabilization of the system.

During the development of the workflow, it was observed that structures obtained directly after the RDKit-based fragment assembly could contain stretched bonds or unfavorable local contacts due to the permissive distance thresholds used during fragment reconstruction. Therefore, a short relaxation stage was incorporated to generate realistic bond lengths and local geometries before comparing the reconstructed poses with the corresponding crystallographic structures. This stage was designed as a structural relaxation step rather than as a conventional molecular dynamics simulation intended to investigate the temporal evolution or equilibrium behavior of the peptide–protein complex.

The relaxation protocol was implemented using GROMACS 2022.3, and only CPU cores with 4 GB of RAM were used. The ligand topologies for each pose were generated by using ACPYPE, supported by an automated preprocessing script. The protein topology was generated using the gmx pdb 2gmx command with the AMBER99SB force field.

The protocol includes a 2 ps energy minimization step (1000 steps), a 4 ps NVT equilibration step (2000 steps), four consecutive 4 ps NPT equilibration steps (2000 steps each), and a final 4 ps simulation run (2000 steps). In total, each assembled peptide–protein complex is subjected to a 26 ps relaxation protocol.

### Post-Processing and Analysis

Several metrics were collected in order to study the accuracy of STELLAR in different kinds of cases. First, for the benchmark cases that contain crystallographic poses, the RMSD is calculated for the complete peptide as well as for each fragment. We used *obrms* for this calculation. For peptide cases, the RMSD is calculated for the carbon backbone of the peptide, whereas for polysaccharides, all atoms are considered.

Energy affinity metrics were also extracted. First, a GNINA affinity score was calculated for each pose using the score-only mode to obtain the energy without performing a new docking calculation. From the MD simulation results, g_mmpbsa^57^ was used to obtain the MM-PBSA energy values for each of the protein–ligand complexes. In addition, the energetic contributions obtained from each fragment docking were saved. Finally, all of these metrics for each valid combination were stored in a single CSV dataset for each case.

### Code Structure

The STELLAR workflow operates within the protocol established by MetaScreener (https://github.com/bio-hpc/metascreener). MetaScreener is an open-source and publicly available tool that enables users to perform molecular docking on high-performance computing (HPC) clusters by using various docking engines, including Gnina. This allows for a significant reduction in overall runtime and makes it feasible to screen large compound libraries. It also facilitates the exploration of the entire molecular surface by splitting it into different grids.

MetaScreener addresses these challenges by abstracting both the use of the docking software and the interaction with the user’s queue manager, providing a simple and unified command-line interface. Additionally, it supports multiple virtual screening approaches, including both structure-based and ligand-based methods. As such, MetaScreener aligns well with the goals of the STELLAR workflow, particularly in its ability to run multiple fragments efficiently.

In this way, MetaScreener was modified to overcome this kind of problem. The *save_pose_CN_coordinates.py* and aggregate_gn_pose_coords.py scripts were added to the MetaScreener modular organization to extract the coordinates of the atoms involved in the bond of the fragments for each pose obtained. These data were also saved together with the rest of the analysis files generated by MetaScreener in the docking step.

The STELLAR workflow implies the execution of a set of different scripts. The first scripts are related to filtering the starting combinations of poses based on the maximum distance between the atoms involved in the bonds. Because of the large number of Euclidean distance calculations needed to evaluate all possible combinations, a C-written code was developed to carry out this task and was integrated with the rest of the scripts written in Python. *filter_fragment_combination.c* and its compiled static library version, *filter_fragment_combinations*, were designed for this purpose.

The steric overlap filter is launched by the *check_overlap_combinations.py* script. This script calls the *overlap* executable located in the MetaScreener folder (MetaScreener/external_sw/overlap/overlap) to calculate all of the volume values needed to obtain the specific overlap value. Each calculated value is then compared to the assigned tolerance threshold, and only the valid poses are retained until the final pair of fragments.

The valid combinations are saved in a new folder, where the selected poses are joined using *merge_all_combinations.py*. Before merging, the PDBQT files were prepared to avoid reading problems by RDKit. After joining and obtaining the file with the assembled polymer, several treatments were applied to relax the structure before generating the topology and running the MD simulation. These steps are performed by *prepare_final_combinations.py* and *fix_zero_charge_atoms.py*.

A GROMACS module is used to carry out the relaxation of the complex structure through a “very short” molecular dynamics simulation. In this case, STELLAR calls this module using *generate_topologies.py* and *run_md_simulations.py* to generate the ligand and protein topologies and to run the MD simulations, respectively.

The postanalysis is performed by different Python scripts, each one extracting a specific metric from each step of the STELLAR pipeline. To calculate the RMSD between each fragment and its corresponding crystallographic disposition and between the final pose and the polymer crystallographic pose, we use *calculate_rmsd_combinations.py* and *calculate_md_rmsd.py*. The *calculate_score_only.py*, *calculate_mmpbsa.py*, and *calculate_fragment_energies.py* scripts obtain the Gnina score-only energy, the MM-PBSA values for each MD simulation, and the energetic contributions for each fragment pose, respectively.

Finally, *merge_all_metrics.py* extracts all of the information and generates a final CSV file containing all of the data collected for each combination and each successfully assembled polymer. All files and generated data are organized in a single directory by using the *organize_workflow_results.py* script.

All of this is embedded in Singularity containers to ensure reproducibility and compatibility across different HPC systems. Figure 2 shows the modular structure of the MetaScreener code with the new functionalities introduced by STELLAR.

**Figure 2:**
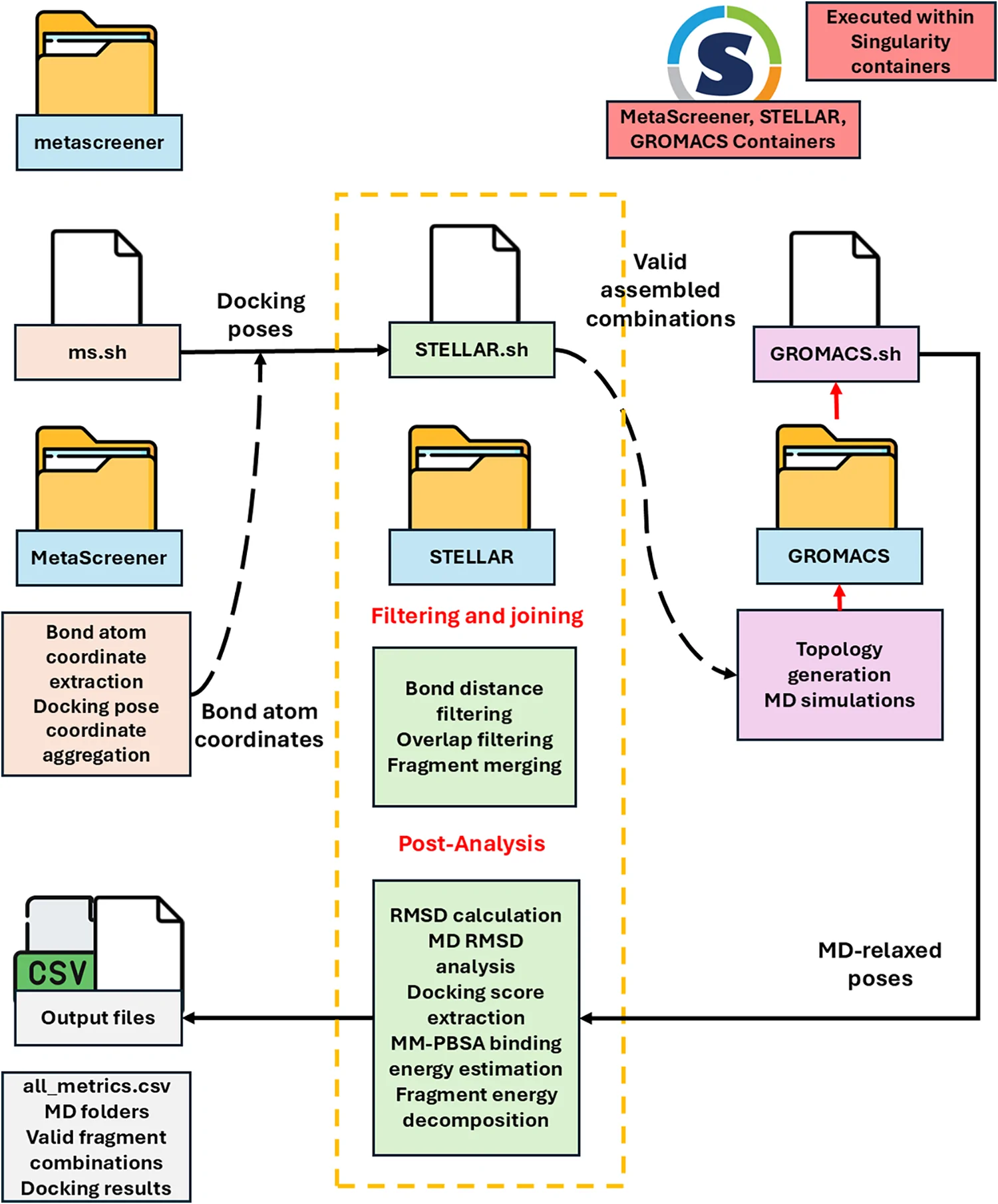
MetaScreener launch script (ms.sh) obtains docking poses for each fragment and extracts the bond atom coordinates. STELLAR filters and assembles compatible fragment combinations based on bond distance and overlap constraints. The resulting peptide assemblies are subsequently refined through molecular dynamics simulations using GROMACS. Finally, a postanalysis module computes structural and energetic metrics, including RMSD, docking scores, MM-PBSA binding energies, and fragment energy decomposition. All modules are executed within Singularity containers to ensure reproducibility and portability across HPC environments.

The organization and structure of the code were supported by AI-assisted programming tools such as Cursor 3.1.14 using Anthropic Claude Opus 4.8 ^58^ and Composer 2.5 ^59^ models.

Due to the low initial assembly rate and the reported gains in productivity and efficiency associated with agentic programming,^60^ we employed an agentic loop to harden the code and improve the robustness of STELLAR.^61^ This automated validate–diagnose–repair–retry procedure was driven by a large language model coding agent (Anthropic Claude Opus 4.8,^58^ via the Cursor SDK^62^): each case was staged in isolation, run with full logging, and, on failure, the failing log and a curated rules document were passed to the agent, which diagnosed the root cause and applied a targeted source-code patch before the case was re-run. A case is counted as successful when the full pipeline produces the consolidated table with at least one pose having a non-missing MD-refined RMSD (rmsd_md). Recurring fixes concerned environment portability, clash-free combination sampling, ligand-topology robustness, and robust RMSD evaluation. All fixes were reviewed and integrated into the standard pipeline, so the improvements are deterministic and reproducible independently of the agent, substantially increasing the assembly rate.

All generated components were systematically validated through unit testing and manually reviewed to ensure the functional correctness. In total, 16 unit test modules were developed to validate the main stages of the workflow, including fragment processing, coordinate extraction and validation, filtering and assembly procedures, overlap detection, molecular dynamics execution, MM-PBSA calculations, metric aggregation, and workflow organization. These tests were designed to verify the consistency of the generated code and to identify potential implementation errors or unintended artifacts before they were incorporated into the final workflow.

### Validation

The STELLAR protocol was tested for a diverse benchmark compiled by peptide–protein complexes obtained from the Propedia database.^49^ The selection was performed considering the greatest possible variety of peptides (chain size and type of amino acids) and the target proteins. When the complexes were chosen for the benchmark, their structures were extracted in PDB format from the PDB database.^63^ To secure a correct run for the calculations using the different docking methods that STELLAR uses, the proteins were extracted from the complete system obtained from the PDB database. Subsequently, the protein structures were processed and exported to MOL2 format via MAESTRO’s Schrödinger software,^51^ adding the hydrogens and charges to the final file. A similar protocol was used to save the protein in the PDBQT structure via AutoDockTools^53^ software, which performed the same tasks and applied the AD type to the atoms.

Each one of the selected complexes was launched in the STELLAR protocol, indicating the prepared protein structure and the sequence of amino acids of the corresponding peptide according to Propedia information. Each one of the calculations generated a table with the results for each cluster, with information about the final docking score and the interactions for each fragment with the residues of the protein.

Finally, each pose of the final peptide obtained was compared with the crystallographic pose extracted from the original PDB file, calculating the RMSD for each one of the predicted poses against the crystal structure of the peptide using the *obrms* tools from OpenBabel Tools.^64^

### Large-Scale Computational Execution

3,390 protein–polymer cases (3,382 protein–peptide and 8 protein–polysaccharide systems) were launched following the entire pipeline developed. A total of 265 complexes (257 protein–peptide and 8 protein–polysaccharide systems) were successfully validated using STELLAR, and their RMSD values were calculated for each one (100 combinations each) in parallel. All executions were performed within a Singularity environment on the BSC Supercomputing Center supercomputer (https://www.bsc.es/).

Docking calculations and STELLAR scripts were executed using the Singularity image metascreener_22.04.simg without GPU support, and jobs requested 4 cores in the internal command parameters. CPU cases (Gnina docking, STELLAR scripts) were run on CPU using 1 node, 1 task, and 1 CPU core per job. Memory was set to 20 GB in the application parameters. SLURM used the default queue, QoS gp_resa, and a wall time of 3 days.

MD simulations and GPU-dependent jobs were executed with the Singularity image gromacs2022.sif with the --nv flag for GPU access, and srun was used to launch the jobs. GROMACS was configured with 24 cores and 1 GPU for non-bonded calculations. SLURM used the GPU partition, QoS acc_resa, and a wall time of 30 min. The memory was set to 4 GB in the application parameters. Threading was limited to 1 via the OMP_NUM_THREADS, MKL_NUM_THREADS, and OPENBLAS_NUM_THREADS.

### Interpretability Machine Learning

In one of the cases tested by STELLAR, the maximum possible number of combinations was generated to obtain an adequate dataset to train a machine learning model and evaluate its capacity to predict new peptides with a high probability of binding in a similar way to the chosen binding site. Additionally, the interpretability obtained from the model predictions was analyzed to obtain an explicative score to rank these new hits. In this case, the p38α complex was selected due to its importance in cancer drug discovery.

The dataset obtained with all valid combinations consisted of 938 valid combinations. Valid combinations were considered those that produced an assembled peptide with an RMSD value relative to the crystallographic peptide pose. These valid combinations correspond to distinct assembled poses generated from different combinations of fragment docking poses within the same protein–peptide system. The dataset contained 68 columns extracted from the different steps of the STELLAR workflow, including the combination ID (*combination_id*) and the RMSD compared to the crystallographic peptide pose (*md_rmsd*). This dataset was used to create different Machine Learning models using several algorithms.

For this objective, the in-house SIBILA software (https://github.com/bio-hpc/sibila) was used.^65^ All available techniques in Sibila were evaluated: K-nearest neighbors (KNN), random forest (RF), decision tree (DT), support vector machine (SVM), XGBoost (extreme gradient boosting), logistic regression (LR), and bootstrap aggregating (Bagging). Machine learning interpretability analyses were also performed for each of these models. The interpretability analysis included Shapley values, LIME, and permutation feature importance methods.

The interpretability data from the models with the best performance and accuracy (evaluated using *R* ^2^ values) were analyzed to infer a formula capable of obtaining an acceptable regression with RMSD relative to the crystallographic pose.

To obtain the final formula, we explored a linear regression model considering the most important features extracted from the interpretability analysis (15 variables) against *md_rmsd* values. First, an OLS linear regression was performed to obtain a standard model with 15 variables. Subsequently, a sweep of combinations of the parameters was performed from 1 to 15 variables until the best R^2^ score was obtained. With the best formula, modifications of the variables (using logarithmic or inverse transformations) and the inclusion of interactions (products between different variables) allowed an optimal regression value to be obtained. This analysis was carried out using a Python script that imports the Pandas, NumPy, and SciPy libraries.

## Results

The validation of the tool was carried out using a benchmark dataset derived from the Propedia database of peptide–protein complexes. From this dataset, the necessary PDB codes were extracted to obtain the corresponding crystallographic structures. Also, 8 polysaccharide cases extracted from the PDB database were tested using the same pipeline with slight modifications.

Each case successfully generated an individual final dataset with metrics and information extracted from the different steps from the STELLAR pipeline. Individual fragment and assembled pose RMSD values compared to crystallographic poses were calculated. The total affinity from Gnina was obtained. These correspond to both the raw value calculated using the Vina scoring function (*total_affinity*) and its subsequent optimization performed by Gnina (*total_score*).

Several values were also collected from the combination of filter steps. The C–N (or other bond involved atom) distance was measured between each one of the adjacent fragment pairs (*distance_frag­(n)_to_frag­(n+1)*). Postrecomposition steps were also considered to extract variables from there, especially after structure relaxation via GROMACS. For each successfully assembled combination, the GNINA affinity score of all of the polymers using *score-only* parameters, MMPBSA from the short MD simulation, and all of the energetic contributions of each fragment from individual docking were saved in the final dataset.

RMSD between each valid assembled peptide and its corresponding crystallographic peptide pose was the fundamental metric to compare the accuracy and performance of STELLAR in this kind of case. First, the MD simulation’s last frame and crystallographic complex were aligned, and the RMSD for the carbon-α backbone was calculated.

### STELLAR Successfully Predicted Docking Poses for 257 Protein–Peptide Complexes

The benchmark prepared for protein–peptide complexes was divided into four different sections. Each section is composed of a set of complexes with a specific range of amino acids in the peptide sequence. In total, the STELLAR benchmark contains 3382 cases, where 1,668 cases contain peptide ligands with 10–12 residues (4 fragments), 822 contain between 13 and 15 residues (5 fragments), 447 contain between 16 and 18 residues (6 fragments), and 445 contain between 19 and 21 residues (7 fragments) (Table 2).

**Table 2:** Number of Cases Launched by STELLAR and Success Rates for the Peptide–Protein Complex Benchmark Ordered by the Number of Fragments

number of fragments	range of residues	run cases	assembled cases	assembly rate (%)
4	10–12	1668	129	7.73
5	13–15	822	62	7.54
6	16–18	447	44	9.74
7	19–21	445	22	4.94

For each case, the same parameter treatment was applied. 2 Å was indicated as the minimum distance of the bond between atoms involved in the peptide bond. 350 Å^3^ was established as a steric overlap threshold between adjacent fragments. 100 final combinations were extracted after running both sequential filters. These combinations were processed in the recomposition and relaxation steps. A final dataset with the 100 combinations and all of the metrics for each combination was generated (*all_metrics_GN_CASE.csv*)

The assembled cases are denominated as those peptides for which STELLAR completes all of the pipeline and where it obtains a final pose with the reconstruction of its fragment, so its RMSD compared to the crystallographic pose can be calculated. Therefore, the assembly rate is referred to as the percentage of the cases tested that are able to ensemble and run the corresponding MD simulation properly, obtaining a final RMSD compared to the crystallographic pose.

These low assembly rates are largely associated with preprocessing and topology generation issues required for the subsequent relaxation stage. To better understand the origin of unsuccessful cases, a traceability analysis of the benchmark was performed, and the main causes of failure are summarized in Table S6. The most frequent problems were related to receptor preparation and subsequent topology generation, including inconsistencies in residue numbering, chain organization, ion handling, incomplete atomic definitions, and failures during ligand parametrization. These issues often caused topology generation errors or premature termination of GROMACS before the relaxation stage could be completed. Therefore, a substantial fraction of unsuccessful cases reflects limitations in the automatic preparation of heterogeneous PDB structures rather than failures of the docking and assembly strategies themselves.

Guided by this failure analysis, the revised STELLAR pipeline was re-evaluated on 500 cases that had failed during the initial execution. Of these, 450 successfully completed the workflow and produced evaluable assembled structures, corresponding to a 90% recovery rate. In the remaining 50 cases, no valid fragment combinations satisfying the distance and overlap filters were identified, and therefore, no assembled structures could be generated. These cases were used only to validate the code’s robustness and were not included in the benchmark results.

Despite the difficulty of the automatic preprocessing of the structure obtained by Propedia, 257 cases were processed successfully, and the final results dataset was obtained, generating all of the metrics during the whole process and the comparison of RMSD with the crystallographic pose.

### Poses Obtained and RMSD Values Compared to Crystallographic Pose

The 257 protein–peptide cases obtained were analyzed to evaluate the accuracy of STELLAR in predicting the real pose among the generated combinations. The lengths of the peptides tested ranged from 10 to 21 residues. Thus, each case generates an extensive number of poses for each fragment, obtained through Gnina docking using the multipose parameter, together with high exhaustiveness and RMSD filtering between poses of the same fragment. This represents a large number of combinatorial spaces.

All pose combinations were subsequently filtered by STELLAR based on bond distance and steric overlap criteria. The valid combinations were then assembled, and their structures were relaxed through the MD simulations. Each valid combination was analyzed individually to obtain the energetic and structural metrics.

Figure 3 shows the final results obtained after the STELLAR pipeline, where the RMSD of each valid combination is calculated with respect to the crystallographic pose previously aligned. The results were ordered according to the lowest RMSD values.

**Figure 3:**
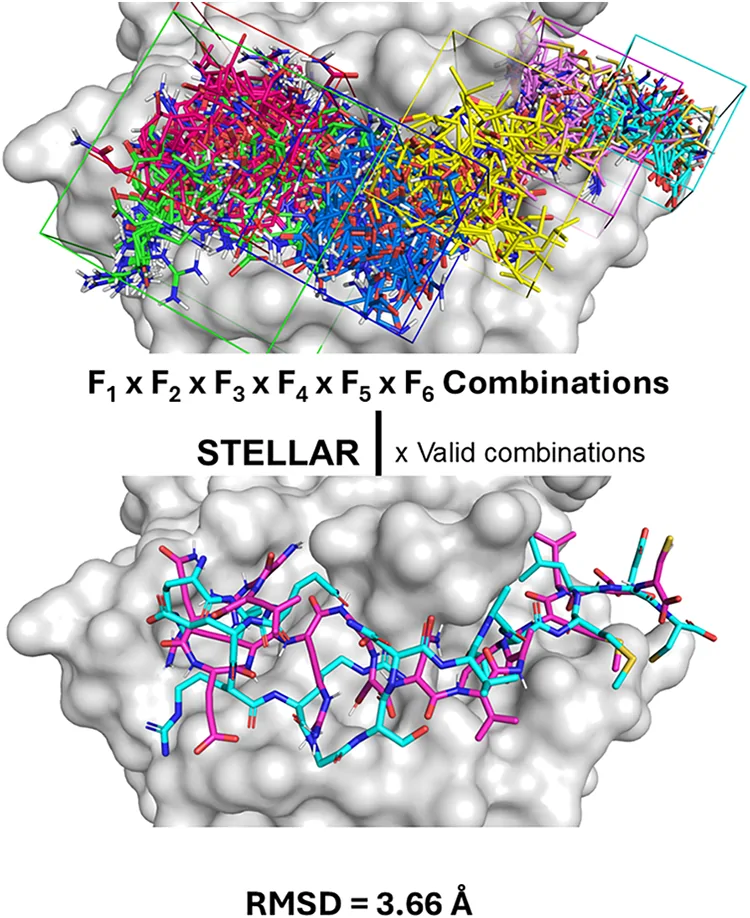
Combination pools generated by Gnina-based docking for the p38α–pepHePTPm complex (PDB ID: 8YP8; peptide length: 13 aa) and the lowest-RMSD valid combination obtained through the STELLAR pipeline. The lowest-RMSD cluster predicted by STELLAR (3.66 Å) is superposed with the crystallographic reference peptide (cyan). The predicted pose (magenta) shows strong spatial agreement within the p38α binding site (deep teal), highlighting the accuracy of STELLAR’s fragment-based reconstruction approach.

The entire process was completed successfully in 257 cases. The RMSD compared to the crystallographic pose was used to check the capacity of STELLAR to predict a realistic pose. An analysis of the accuracy of the methods was performed for each subset of complexes ordered by the comments in the previous section (Figure 4).

**Figure 4:**
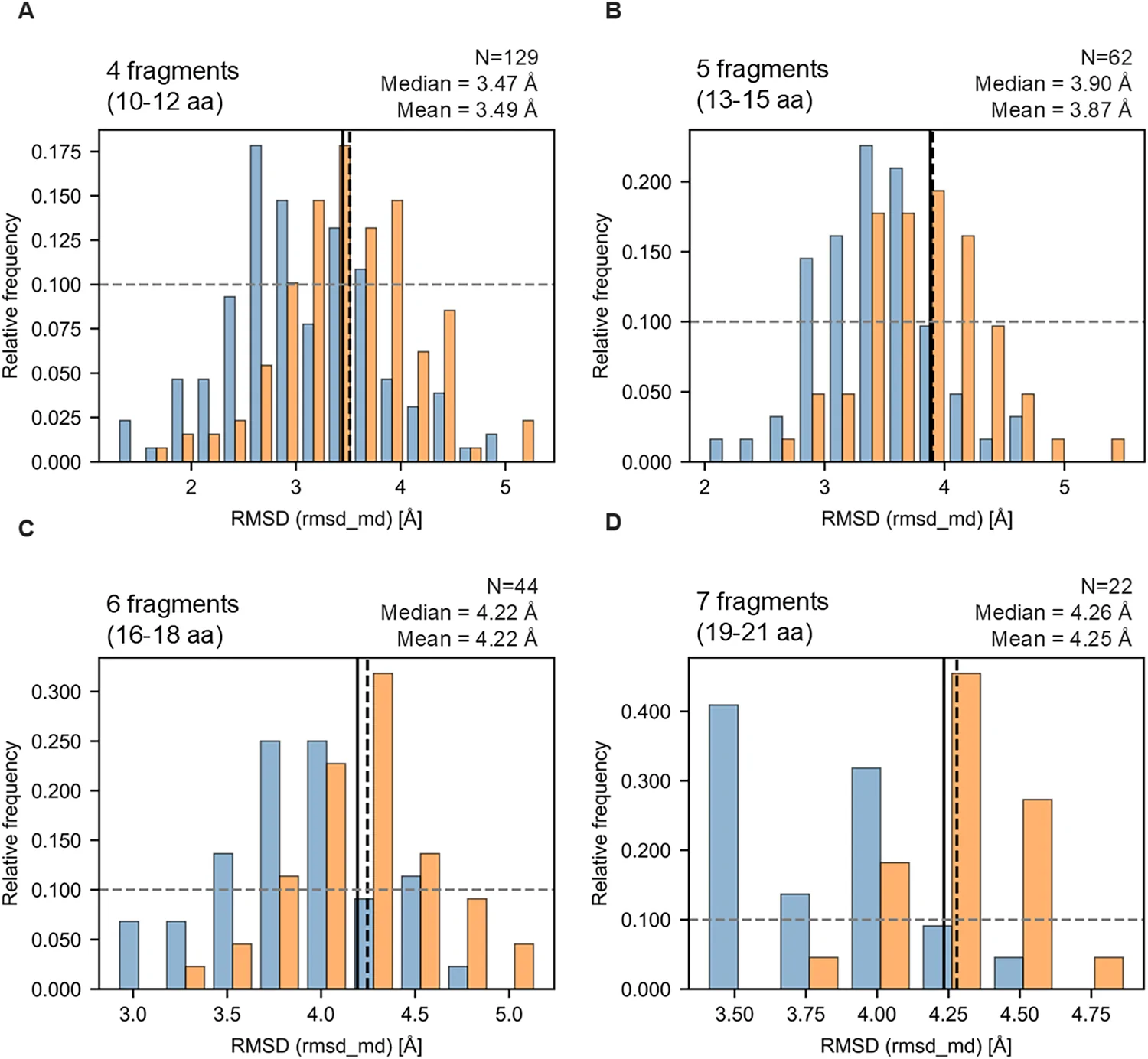
Histogram showing the frequency distribution of minimum RMSD (blue) and mean RMSD (orange) values relative to the Cα backbone of the crystallographic peptide pose. Each panel corresponds to complexes containing peptides with 4 (10–12 amino acids) (A), 5 fragments (13–15 amino acids) (B), 6 fragments (16–18 amino acids) (C), and 7 fragments (19–21 amino acids) (D), respectively. The solid black line indicates the global median RMSD, while the dashed black line indicates the global mean RMSD, both calculated from all individual RMSD_md values across the subsets.

The RMSD values were analyzed for peptides split by 4, 5, 6, and 7 fragments, respectively. The subset of 4 fragments was the one with the highest number of successful cases (*N* = 129). The median RMSD value was 3.47 Å, and the mean was 3.49 Å. The range of RMSD values was between 1.29 Å and 4.97 Å. Regarding the mean RMSD for each case, the range changed to 1.71 Å to 5.19 Å. The region with the highest number of cases, considering both minimum RMSD and mean RMSD, included 22 cases (relative frequency = 0.18) in both scenarios and corresponded to a minimum RMSD range between 2.54 Å and 2.80 Å, and between 3.29 Å and 3.55 Å for the mean RMSD (Figure 4A).

The benchmark subset for peptides split into 5 fragments also showed similar values, with a total of 62 successful cases. In this case, the minimum value for the minimum RMSD was 2.02 Å, while the maximum value was 4.63 Å. The highest population of cases was found between 3.27 Å and 3.52 Å for the minimum RMSD, with 14 cases (relative frequency = 0.23). For the mean RMSD values, the range between 3.77 Å and 4.02 Å included 12 cases (relative frequency = 0.19) (Figure 4B).

Due to the higher complexity and number of rotatable bonds, the subsets corresponding to 6- and 7-fragment peptides showed fewer completed runs, with 44 and 22 successful cases, respectively. The RMSD values obtained were also slightly higher than those for peptides with a lower number of residues. The 6-fragment cases showed a range between 2.90 Å and 4.70 Å for minimum RMSD and between 3.2 Å and 5.1 Å for mean RMSD. For the 7-fragment cases, ranges between 3.38 Å and 4.47 Å for minimum RMSD and between 3.87 Å and 4.7 Å for mean RMSD were obtained (Figure 4C,4D).

Finally, a comparative analysis between the four subsets of protein–peptide complexes and the complete benchmark of 257 cases was performed to evaluate the efficacy and accuracy of the method as a function of peptide length (Figure S4). The mean and median values of the total benchmark, considering the minimum RMSD for each case, were 3.35 Å and 3.45 Å, respectively. For the mean RMSD per case, the mean was 3.76 Å and the median was 3.82 Å. Tables S3 and S4 extend the analysis by reporting additional statistical metrics, including standard deviations and confidence intervals.

The fact that the mean RMSD value is slightly lower than the median suggests a left-skewed distribution, where a subset of low-RMSD, near-native predictions pulls the mean below the median. This indicates that a relevant proportion of the evaluated cases achieved clearly sub-3 Å RMSD values.

Most of the cases were found in the range between 3 and 4 Å (51.36%) for the minimum RMSD. When considering the mean RMSD instead of the minimum RMSD, the highest percentage was also observed in the range between 3 and 4 Å (39.81%). In addition, the proportion of cases with a mean RMSD between 4 and 5 Å increased to 36.58%, whereas in the minimum RMSD metric, this percentage was 17.51%. Overall, most cases showed RMSD values below 4 Å, with 82.49% for minimum RMSD and 61.09% for mean RMSD, while only 2.33% of cases exceeded 5 Å.

For completeness, additional success rates calculated using RMSD thresholds of 2.0 Å, 2.5 Å, 4.0 Å, and 5.0 Å are reported in Table S5. As expected, the proportion of cases below the more stringent 2.0–2.5 Å thresholds decreases as peptide length and the number of fragments increase. This behavior is consistent with the increased conformational complexity associated with highly flexible peptides comprising 10–21 residues. Nevertheless, most benchmark cases remain below 4.0 Å, supporting the ability of STELLAR to recover structures close to the crystallographic pose even for challenging long-peptide systems. For this reason, a 5.0 Å threshold was selected as the primary success criterion throughout this study.

### Runtime Performance of STELLAR Compared to Previous Tools

The improvements introduced by STELLAR represent significant methodological advancements. First, the docking workflow demonstrates a clear reduction in execution time and file processing compared with existing tools. This performance gain is not limited to computational efficiency; STELLAR also improves the accuracy of docking predictions by effectively identifying clusters with high-quality scoring functions that correlate well with experimentally validated structures. This represents a substantial advance for fragmentation-based peptide-docking approaches (Table S7).

Each STELLAR run was configured to generate 100 combinations in order to identify poses with the lowest possible RMSD relative to that of the crystallographic structure. STELLAR enables extensive parallelization of its workflow, allowing docking, molecular dynamics (MD) simulations, and RMSD calculations to be executed concurrently. This strategy facilitates the exploration of a large combinatorial space without a proportional increase in the processing time per combination.

The benchmark was executed in a massively parallel fashion. However, individual complexes were processed sequentially within single SLURM jobs, enabling large-scale parallel execution across the 3382 protein–peptide benchmark cases.

To evaluate the computational performance, execution times were analyzed for representative cases covering different peptide lengths. Specifically, complete STELLAR runs (100 combinations) were evaluated for PDB entries 2ND1 (5 fragments) and 3R7G (7 fragments), representing the lower and upper bounds in computational cost. Additionally, single-combination executions were analyzed to assess the intrinsic cost of processing the individual poses.

Execution time increased significantly when processing 100 combinations compared with a single combination, primarily due to the increased number of MD simulations and subsequent analysis steps. The overall execution trends remained consistent with single-combination runs (Figure S5), with MD simulations representing the main computational bottleneck.

A comparison between peptides split into 5 fragments (Figure S6) and 7 fragments (Figure S7) revealed a substantial increase in total execution time, from 7.18 to 17.79 h. The main contributors to this increase were docking (1.16 vs 1.47 h) and, more notably, MD simulations (4.98 vs 15.15 h). Additional post-processing steps, including RMSD and energy calculations, also showed moderate increases.

A detailed analysis of the 3R7G case confirmed that most of the computational time is dominated by docking and MD simulations. Docking, performed with high exhaustiveness parameters, required 1.47 h across all runs, while MD simulations accounted for the majority of the total runtime. Steric overlap filtering also contributed significantly (0.53 h), whereas other steps remained below 100 seconds per task.

Single-combination executions exhibited a near-linear scaling of runtime with respect to the number of fragments (Figure 5). The total execution time increased from 1.56 h (4 fragments) to 2.27 h (7 fragments), suggesting good scalability for larger peptides beyond the tested range.

**Figure 5:**
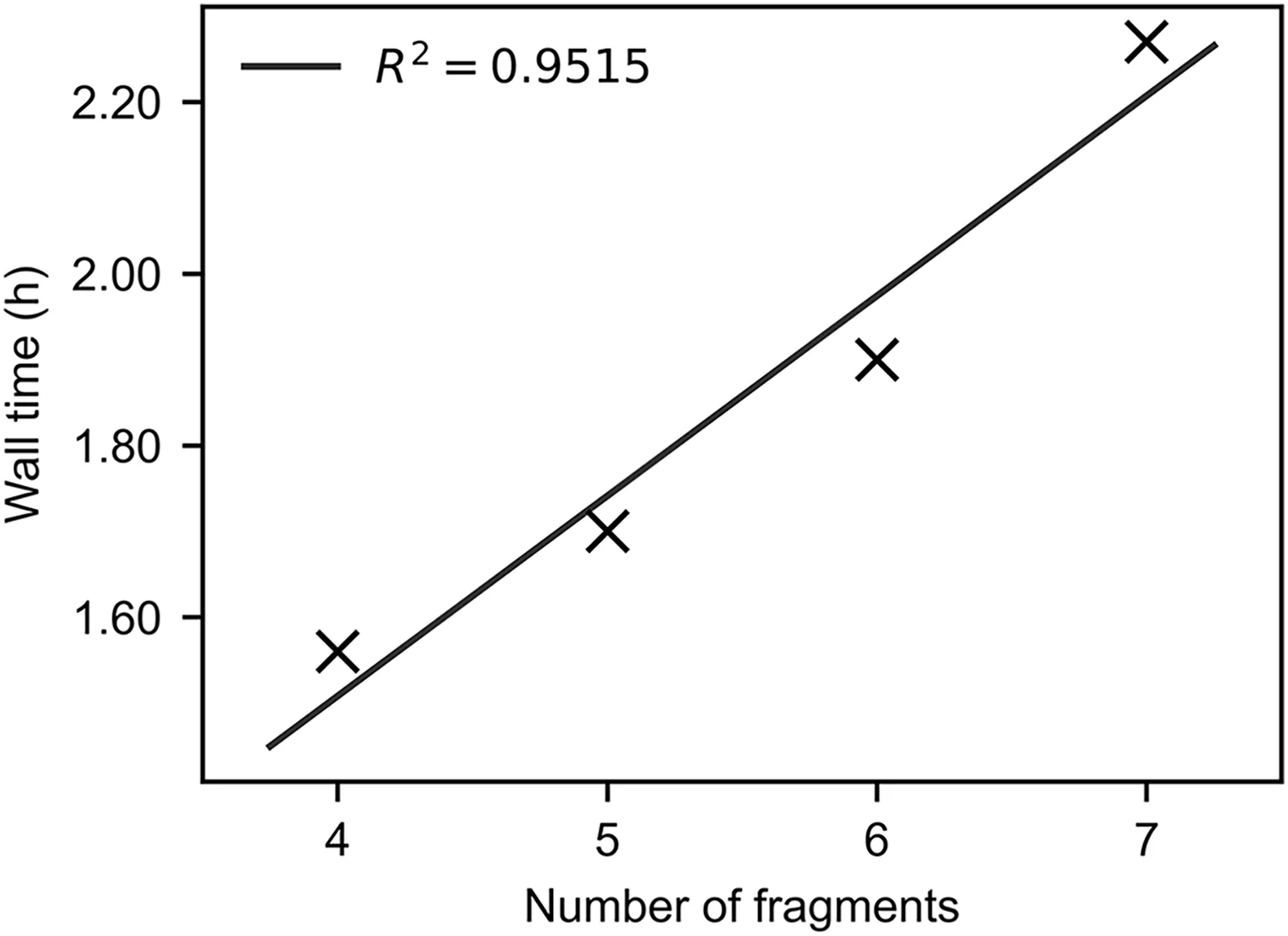
Wall-clock runtime as a function of the number of fragments. Crosses denote measured runtimes for 1J4Q (4 fragments), 2ND1 (5), 1BXL (6), and 3R7G (7). Runtime increases approximately linearly with each additional fragment.

Overall, STELLAR enables the generation of a large number of valid combinations while maintaining an efficient execution through parallelization. This enables efficient exploration of large conformational spaces for highly flexible peptides, extending the applicability of docking workflows to longer peptide systems than those commonly reported in the literature. These improvements position STELLAR as a high-performance and scalable platform for large-scale peptide-docking studies.

STELLAR is capable of generating a large number of valid combinations simultaneously. Through efficient parallelization, the workflow achieves performance comparable to or exceeding that of existing tools, even when handling longer peptides and multiple poses. These improvements enable STELLAR to significantly accelerate the exploration of the conformational space while maintaining a near-linear increase in computational cost with respect to the number of fragments. As a result, STELLAR represents a high-performance and scalable platform for peptide-docking applications.

### Exploratory Analysis of the Binding Mechanism between the HePTP KIM Peptide and p38α D-Recruitment Site

One of the main objectives of this work was to develop a robust docking workflow capable of ranking and predicting new candidates involving large chains such as peptides and polysaccharides. Because of their high number of rotatable bonds, these types of polymers are computationally expensive and remain a significant challenge for conventional docking approaches, often leading to the exclusion of this large chemical space.

STELLAR achieves low RMSD values across the different peptide–protein benchmark cases, provided that at least one assembled combination is obtained. After evaluating STELLAR’s ability to reproduce poses close to the crystallographic reference, a more in-depth analysis was conducted to identify variables and features that positively influence the attainment of competitive RMSD values compared to those reported in the literature. Based on this analysis, the final goal was to establish new guidelines for specific cases that enable efficient and accurate ranking of multiple modifications of the peptide crystal sequence.

In this context, the crystallographic complex of the HePTP KIM peptide bound to the p38α D-recruitment site was studied in depth (PDB: 8YP8). The D-recruitment site (CD/ED domain) of p38α has been relatively underexplored, mainly due to the challenges associated with protein–protein interactions. However, this site plays a fundamental role in numerous p38α-mediated protein–protein interactions and is involved in multiple downstream regulatory pathways. Targeting this region provides an alternative strategy to modulate the activity of the enzyme without directly inhibiting the catalytic site.^66^

Identifying peptides capable of reproducing this specific interaction offers new opportunities for the development of p38α inhibitors, which are widely investigated in cancer therapy. In the selected case, the peptide corresponds to the KIM region of HePTP, which specifically interacts with the CD/ED domain of p38α. This peptide acts as a regulatory element that modulates the interaction between HePTP and p38α. The adjacent regions define the docking epitope and can serve as templates for the design of peptide mimetics and novel allosteric inhibitors.^67^

The identification of new compounds, such as peptides that perturb docking interactions, may enable allosteric inhibition or selective modulation rather than complete catalytic blockade, representing a promising avenue for future research.

These types of interactions are particularly delicate, as they must be carefully designed to perturb specific protein–protein contacts without disrupting essential regulatory interactions. Therefore, the study of new peptide candidates targeting this region, while preserving the crystallographic binding mode, represents a promising strategy for the development of novel p38α inhibitors.

For this reason, the binding of this peptide to the D-recruitment site was investigated by using STELLAR. In this case, the standard filtering step limiting the analysis to 100 combinations was omitted in order to maximize the number of fragment combinations explored and identify the key metrics influencing RMSD optimization in the final assembled peptide. This analysis is directly related to the identification of alternative peptides with similar binding modes.

The final dataset for this complex comprised 1563 valid combinations after applying distance and overlap filters. Following the assembly and molecular dynamics (MD) simulation steps, 938 combinations (assembly rate = 60.01%) yielded a valid assembled peptide with an RMSD value relative to that of the crystallographic pose. This dataset of 938 combinations includes all metrics generated throughout the STELLAR pipeline (68 features). It was used to train predictive models using various machine learning (ML) and deep learning (DL) approaches, including an additional interpretability layer. This interpretability framework provides valuable insights into the most relevant variables for predicting the realistic binding poses and mechanisms.

### Training Interpretable Machine Learning Models Using STELLAR Outputs

One of the main challenges in peptide docking is not only generating plausible poses but also identifying which of the generated candidates is most likely to resemble the native binding mode. As shown in the previous section, conventional docking scores obtained from GNINA exhibited almost no correlation with the crystallographic RMSD values (*R* ^2^ = 0.015) for the p38α–HePTP system. Therefore, we explored whether the information generated by STELLAR during docking, assembly, and relaxation could be used to derive an interpretable ranking metric capable of prioritizing candidate poses.

To investigate this possibility, several machine learning models were trained using the descriptors generated by STELLAR, and their predictions were subsequently analyzed by using interpretability techniques to identify the variables most strongly associated with pose quality. The objective of this analysis was not to develop a universal scoring function but rather to demonstrate that STELLAR outputs can be exploited to generate system-specific ranking models that improve pose prioritization.

The dataset generated by STELLAR for the p38α–HePTP KIM peptide complex was used as input for model training. All machine learning techniques available in Sibila were evaluated, and a model was trained for each method. The most promising approaches, based on the coefficient of determination (*R* ^2^), were Bootstrap Aggregating (BAG), Random Forest (RF), and XGBoost.

In the case of BAG, the final model achieved an *R* ^2^ value of 0.845 (Figure S8), indicating a strong correlation between predicted and true values. RF exhibited the highest predictive performance, reaching an *R* ^2^ value of 0.88 (Figure S9). XGBoost also produced a highly accurate model (Figure S10); despite achieving a slightly lower *R* ^2^ than the other two approaches, it was still considered suitable for evaluating predictive performance and interpretability.

Given that all three models achieved high *R* ^2^ values, a consensus analysis of the most relevant variables across the three methods was performed. This approach aimed to identify common patterns and derive general rules to predict fragment combinations, leading to low RMSD values.

The remaining models (k-nearest neighbors (KNN), decision tree (DT), support vector machine (SVM), and logistic regression (LR)) showed limited predictive capability, with *R* ^2^ values below 0.7. Consequently, they were excluded from further interpretability analysis and from the derivation of predictive rules for identifying peptides with strong agreement with the crystallographic RMSD.

### Machine Learning Interpretability Analysis Identifies Key Features in the p38α D-Recruitment Site Binding Mechanism

The models trained using BAG, RF, and XGBoost were selected for the interpretability analysis due to their high predictive performance. The main objective of this analysis was to identify the most important features contributing to each model in order to better understand the binding mechanism and to derive a predictive framework capable of identifying fragment combinations that closely reproduce crystallographic poses.

Furthermore, this framework could be applied to future docking studies targeting the same site, but involving peptides with different sequences and identical fragment lengths. This approach enables the identification of novel peptide candidates with similar interaction patterns, which may be considered potential p38α inhibitors.

Feature importance was analyzed independently for each model by using multiple interpretability techniques. SHAP (SHapley Additive xPlanations) analysis was used to identify the most relevant features contributing to the prediction as well as their positive or negative impact on the predicted md_rmsd values. For SHAP interpretation, features were considered significant when their distributions showed a clear separation between positive and negative contributions with consistent clustering of high or low feature values within each group.

In the LIME analysis, features with an average attribution below 10^–3^ were considered to have a negligible impact on model predictions. Similarly, in permutation importance analysis, features with importance values greater than 0.05 were considered to have a significant impact on the model performance.

In the BAG model, frag1_Hydrogen_Bonds showed a markedly higher importance compared to the rest of the variables. According to SHAP analysis, *frag1_Hydrogen_Bonds* reached a value of 18.374, clearly dominating the feature contribution. High feature values are consistently associated with higher RMSD values and exhibit a positive SHAP impact (0.1 to 0.2), whereas low feature values correspond to lower RMSD values and show a negative SHAP impact (−0.2 to −0.1). The next most important variables identified by SHAP were *frag3_score* (4.445) and *frag2_Gauss1* (4.139) (Figure S11). Both LIME (Figure S12) and permutation feature importance (Figure S13) analyses further confirmed the dominant role of frag1_Hydrogen_Bonds, showing a clear gap compared to the remaining variables, with an average attribution of 1.3 × 10^–2^ and an importance value of 0.5.

Interpretability analysis of the RF model also identified *frag1_Hydrogen_Bonds* as a key variable, although with a more balanced distribution of feature importance compared to that of the BAG model. In the SHAP analysis, *frag1_Hydrogen_Bonds* (6.567) exhibited a consistent pattern where low RMSD values were associated with negative SHAP contributions and high RMSD values with positive contributions. In addition, *distance_frag1_to_frag2* (4.691), *frag1_score* (4.562), and *frag1_Gauss2* (4.422) also showed significant contributions with clear separations between high and low feature values (Figure S14). LIME analysis revealed a broader distribution of relevant features, with several variables presenting average attribution values above 10^–3^ (Figure S15). In permutation feature importance analysis, *frag1_Hydrogen_Bonds* remained the only feature exceeding the 0.05 importance threshold (0.075), although other variables such as *frag1_score* (0.045) and *distance_frag1_to_frag2* (0.043) showed comparable contributions (Figure S16).

In the XGBoost model, a similar pattern was observed among the most important features identified through interpretability analyses. frag1_Hydrogen_Bonds exhibited the highest SHAP attribution value (23.27), with a strong and consistent relationship between feature values and md_rmsd. High feature values were associated with higher RMSD values, while low feature values corresponded to lower RMSD values. Other features, such as *frag2_Gauss1* (10.038), *frag2_score* (6.515), and *frag3_score* (5.904), also showed notable contributions. However, unlike frag1_Hydrogen_Bonds, they did not display a clearly defined trend in the SHAP distributions (Figure S17). The *frag1_Hydrogen_Bonds* feature also dominated the feature importance rankings in both LIME (Figure S18) and permutation feature importance analyses (Figure S19), with an average attribution of 1.4 × 10^–2^ and an importance value of 0.88, respectively.

By integration of the results from the three interpretability methods across the BAG, RF, and XGBoost models, a total of 15 variables were identified as consistently relevant. These features were subsequently selected to derive a predictive framework aimed at correlating fragment combinations with RMSD values relative to those of the crystallographic pose.

### Rules Derived from Key Model Variables Yield a Formula Highly Correlated with RMSD Values

A considerable number of tools have been developed to dock large molecules, such as peptides, using a variety of approaches. However, the ability to define reliable ranking metrics capable of accurately evaluating the resulting poses remains a significant challenge and has not yet been consistently achieved across existing software and pipelines reported in the literature.

To assess this limitation, the RMSD values calculated for the valid combinations obtained in the p38α–peptide case were compared against their corresponding docking scores generated by a standard method (Gnina, using the score-only protocol). The resulting correlation yielded an *R* ^2^ value of 0.015, indicating a complete lack of correlation between the two variables (Figure 6A). These results highlight the limitations of conventional docking scoring functions in accurately reflecting the true binding affinity and structural accuracy of peptide poses relative to their crystallographic reference.

**Figure 6:**
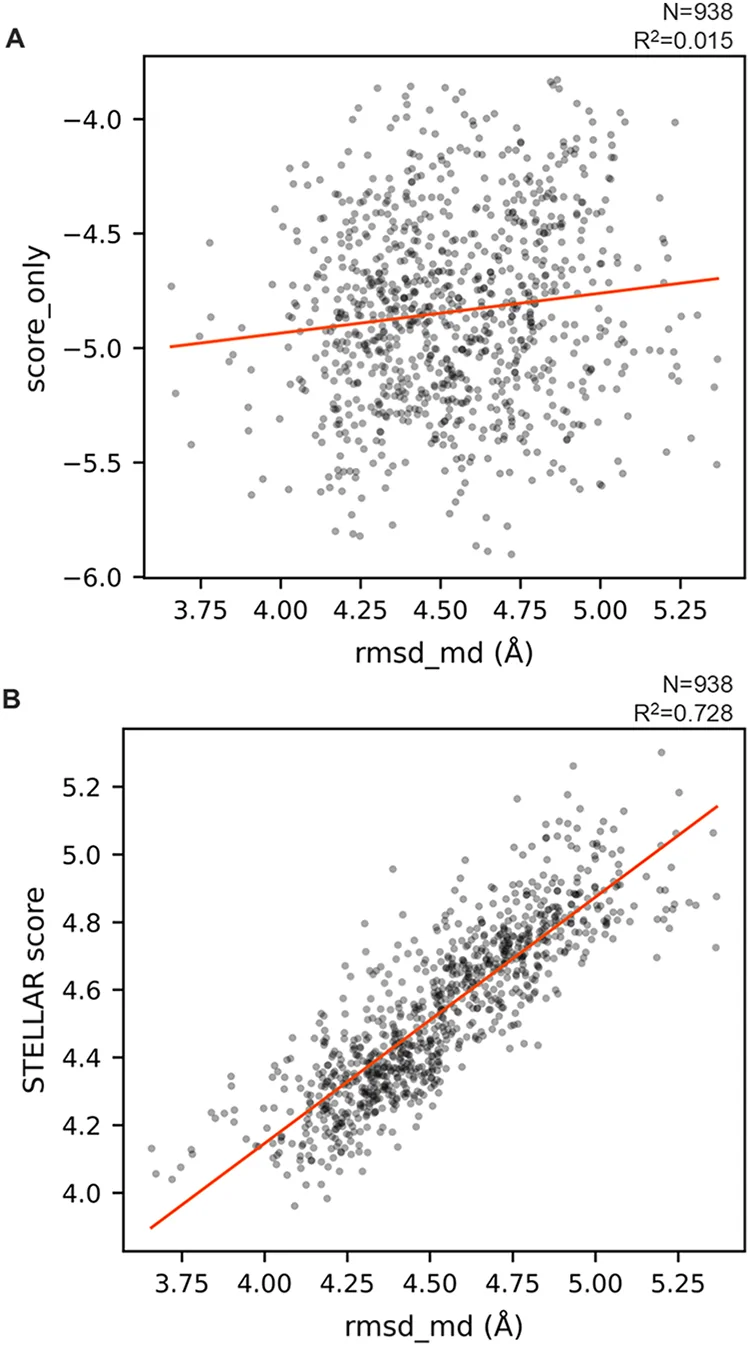
Scatter plots of 938 valid assembled combinations for the p38α–HePTP KIM peptide complex, showing the relationship between RMSD_md and score_only (A) or the STELLAR score (B). While score_only exhibits negligible correlation with RMSD_md (*R* ^2^ = 0.015), the STELLAR score shows a strong correlation (*R* ^2^ = 0.728), enabling improved discrimination of near-native conformations.

To address these limitations, a dataset composed of valid peptide fragment combinations generated by STELLAR was constructed, incorporating multiple metrics to characterize each assembled peptide. Subsequently, an interpretability-driven machine learning analysis was performed, leading to the identification of 15 key variables. These variables were used to derive an interpretable ranking model for the p38α–HePTP system, aimed at improving the prioritization of low-RMSD poses relative to conventional docking scores.

A least-squares fitting procedure was then applied to these 15 variables in order to derive a predictive formula showing a strong correlation with RMSD values relative to the crystallographic reference.

Additionally, transformations of selected variables were explored to further enhance the correlation. In particular, MMPBSA-related terms were investigated because of their sensitivity and tendency to exhibit larger fluctuations compared to traditional scoring functions such as those implemented in Gnina. Among the tested transformations, applying a logarithmic transformation to the *MMPBSA_elec* component resulted in the most significant improvement in correlation, as measured by the R^2^ metric.

The fragment distance was another variable selected for transformation. In this case, representing the distance as its inverse improved the model performance. Shorter distances between fragments are typically associated with more stable assembled peptides and poses closer to those of the native crystallographic structure. Therefore, lower distance values should contribute more strongly to achieving a higher similarity with the reference pose.

Additionally, five interaction terms between selected variables were incorporated into the proposed formula: *frag1_Hydrogen_Bonds × frag1_Total_Affinity, frag1_Gauss2 × frag1_Total_Affinity*, *frag3_score* × *frag3_Hydrogen_Bonds*, *frag3_score* × *frag3_Gauss2*, and log­(*−mmpbsa_elec_raw*) *× mmpbsa_tota*l. These interaction terms were identified through a systematic exploration of variable transformations aimed at optimizing the model and maximizing the R^2^ value.

The p38α-specific STELLAR ranking model was ultimately defined as a linear model including these pairwise interaction terms, with model coefficients reported in Table S1. When applied to the dataset, this scoring function achieved an R^2^ value of 0.728 with respect to RMSD (Figure 6B).

Within this specific protein–peptide system, the derived ranking model showed a substantially stronger correlation with RMSD than the Gnina score, highlighting the potential of STELLAR-derived descriptors for pose ranking.

### STELLAR Successfully Applies to Carbohydrate–Protein Complexes

STELLAR demonstrated consistent results with low RMSD values compared to crystallographic peptide poses in different protein–peptide complexes. However, peptide–protein docking is a widely explored field. To demonstrate the applicability of the STELLAR pipeline to other types of large polymers, 8 linear carbohydrate–protein complexes were evaluated using a slightly modified version of the STELLAR code.

The critical point in these cases was to correctly assign the atoms involved in the specific bond. One case with a 1 → 3 glycosidic bond (1GUI) (Figure 7A) and seven cases with 1 → 4 glycosidic bonds (1EO7, 1FFR, 1EIB, 1EHN, 1G9J, 1CXK, 1E0O) (Figure 7B) were evaluated. Each case required the definition of specific parameters for this purpose.

**Figure 7:**
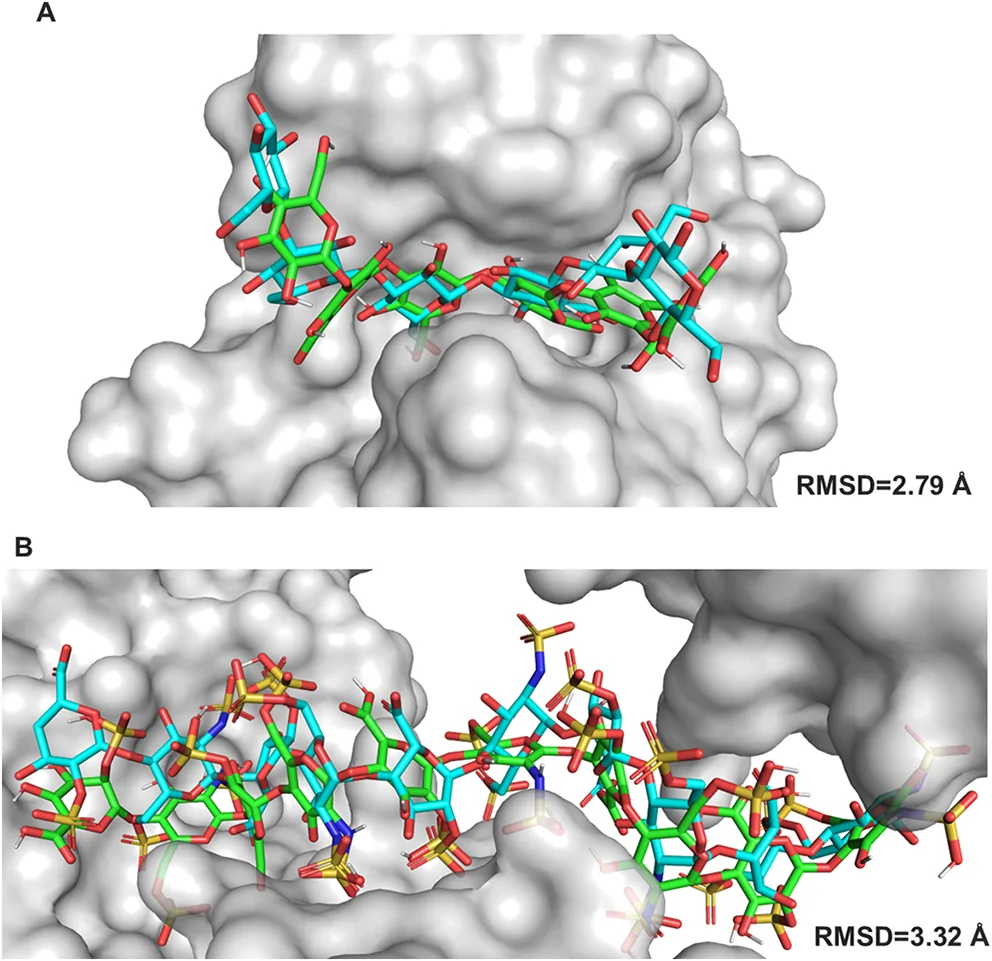
Assembled polysaccharides with the lowest RMSD values generated by STELLAR (green), compared to the crystallographic pose (cyan), for the 1GUI (6 subunits) (A) and 1E0O (10 subunits) (B) complexes. The lowest RMSD values obtained were 2.79 Å and 3.32 Å, respectively.

The minimum RMSD values ranged between 2.1 and 3.56 Å. Considering only the minimum RMSD values, all eight cases showed values below 4 Å. For the mean RMSD values obtained from all valid combinations in each case, all means were below 5 Å. This is also reflected in the mean and median values. The mean was 2.92 Å, and the median was 3.01 Å for the minimum RMSD analysis. For the mean RMSD values, the mean and median were 4.11 Å and 4.33 Å, respectively.

Table 3 shows the minimum RMSD values obtained for each assembled carbohydrate by STELLAR compared to the crystallographic carbohydrate pose. Each case corresponds to a specific type of carbohydrate with different subunits and fragment compositions. The lengths of the polysaccharides ranged between 6 and 10 monosaccharides.

**Table 3:** Minimum RMSD Values between STELLAR-Predicted Peptide Poses and Their Corresponding Crystallographic Structures for a Diverse Set of Polysaccharide–Protein Complexes: Results Are Reported for Peptides Ranging from 6 to 10 Monosaccharides in Length, Demonstrating the Protocol’s Effectiveness across a Wide Size Spectrum^a^

number of monosaccharides	PDB Code (carbohydrate chain)	sequence	minimum RMSD (Å)
6	1EO7 (B)	(Glcα1 → 4)6	2.69
6	1GUI (B)	(Glcβ1 → 3)6	2.79
7	1FFR (B)	(GlcNAcβ1-4)7	3.56
8	1EIB (B)	(GlcNAcβ1 → 4)8	3.22
8	1EHN (B)	(GlcNAcβ1 → 4)8	3.43
8	1G9J (B)	[Glcβ1 → 4-4ThioGlcβ1 → 4]4	2.10
9	1CXK (C)	(Glcα1 → 4)9	2.22
10	1E0O (E)	[IdoA2Sα1-4GlcNS6Sα1-4]5	3.32

a All RMSD values were computed after full pose reconstruction and alignment against reference PDB entries.

## Discussion

STELLAR represents a novel approach with improvements over molecular docking methods described in the literature. STELLAR also introduces significant enhancements to address the specific challenges associated with simulating interactions among biopolymers. This tool has been tested successfully in peptides and polysaccharides. Through an improved computational architecture and novel algorithms, STELLAR aims to provide a robust and efficient solution for the systematic exploration of potential binding modes, which is crucial for the discovery of new bioactive compounds in a pharmacological context. Thus, STELLAR is positioned as a novel methodology that promises to accelerate and enrich the discovery and validation processes of new bioactive compounds derived from long-chain biopolymers.

The innovation introduced by STELLAR lies in its ability to process long chains of polymers that have traditionally been out of reach for classical docking tools, many of which are limited to fragments of just a few subunits. Its generalizable methodology opens new avenues for the discovery of bioactive compounds by enabling the atomistic simulation of complex interactions that were previously inaccessible. Moreover, the computational efficiency of the pipeline, designed for high-throughput screening and compatibility with HPC environments, reduces the resource demands and accelerates the overall discovery process.

Thus, the use of a fragment-based docking (FBD) strategy, together with a pipeline of scripts to filter and relax the final structure, marks a substantial advancement in modeling mid- to large-sized polymeric chains, establishing a clear methodological novelty within the current landscape of biotechnology with high efficacy.

While the current implementation focuses on peptide–protein interactions, the flexibility of the STELLAR framework allows replicating the process to other biopolymers facing similar computational challenges. This adaptability enhances its potential utility across a broader spectrum of protein–biopolymer systems. The efficacy of this kind of polymer was checked in polysaccharides with two different kinds of bonds (1 → 4, 1 → 3)

STELLAR enables accurate and efficient docking of large peptides (10–21 amino acids) and polysaccharides (6-10 monosaccharides), offering significantly better performance than existing tools in terms of scalability, speed, and structural fidelity (Tables S7 and S8). In terms of RMSD, considering a success case as those cases where at least a combination is fewer than 5 Å, 100 % of the minimum RMSD cases are successful, and more than 95 % in all of the cases can be considered successful cases in all of the peptides assembled in a proper way (Table S2).

Polysaccharide cases proved to be particularly successful, yielding especially low RMSD values. This may be explained by the nature of these polymers, which are typically composed of simpler repeating units and may exhibit more constrained bonding patterns compared to amino acids and their peptide linkages.

Besides obtaining a pipeline capable of replicating with accuracy the binding poses of different biopolymers, STELLAR has also conceived a way to rank with an accurate metric these kinds of polymers. This feature allows the use of these tools in the exploration of new hits as in different contexts. With this objective, we perform the test with the complex p38α KIM peptide, a peptide that is composed for the specific site of the protein HePTP that binds specifically to the CD/ED site of p38α.

A dataset with all of the combinations of fragments and the valid assembled peptides was used. Each combination has each one of the metrics that STELLAR has collected during the whole process (from docking, distance filtering, and MD simulations). An interpretability analysis was performed for each of the generated models. BAG, RF, and XGBoost methods generated training models from this dataset, obtaining three of them with a high predictive value. The variable with the most impact, according to the interpretability results, was studied to understand the features when candidate peptides had to be selected.

For each of these models, the variables detected with high impact were studied in depth. The most important feature with respect to the other variables in the three models analyzed was *frag1_hydrogen_bonds*. These values correspond to the energy contributions obtained from Gnina, related to the hydrogen bond interactions between the CD/ED site and the first fragment split from the whole peptide. The sequence corresponding to this fragment is composed of an arginine, a leucine, and a glutamine (R16, L17, and Q18).

The importance of the fragment 1 in simulating a realistic pose for the crystal structure of 8YP8 can be related to the binding mechanism involved in the CD/ED site and the HePTP KIM site. Interaction analysis performed by PLIP in the crystal structure detects several hydrogen bond-based interactions between R16 and Q18 and different residues in the p38α CT/ED site (Figure S20). Strong interactions in the R16 residue to Tyr132 y Tyr311, with a distance between H-A 2 to 2.2 Å and donors angle with 146° and 163°, were detected by PLIP. Gln18 also shows direct contacts related to hydrogen bond interactions, binding to Asp161 and Glu163.

Biochemical and structural studies also suggest the importance of the first fragment of the HePTP KIM peptide in binding, acting as an initial anchoring point that properly orients the KIM motif within the CD/ED site of p38α. Arg16 establishes favorable interactions with residues of this binding site, in line with previous structural studies, while Gln18 also contributes additional contacts that reinforce the interaction. Altogether, these hydrogen bond-based interactions favor a more stable and specific binding of the peptide to the surface of p38α.^68,69^

This could suggest that the hydrogen-bond-based interactions in the R16 and Q18 residues from the first fragment of the peptide play a key role in the specific binding between the complete peptide and the CD/ED site. This is aligned with the hypothesis obtained by the different models, where *frag1_Hydrogen_Bonds* is an important feature to obtain a realistic pose of the peptide via docking. This area can be key to the specific binding in this part of the protein. The information obtained from this study could be useful to develop modifications of this peptide that act as mimetic peptides with allosteric inhibition activity.

Another metric with a strong impact on the predictive model was the distance between the adjacent fragments. Considering fragment proximity as a robust criterion for properly assembling the peptide, minimizing distances of the peptide bonds is a key factor in improving similarity to the crystal pose, which may explain the influence of these variables on the prediction.

Regarding the importance of each fragment in the final peptide, the contributions of the fragments 1, 2, and 3 were more significant when the model predicted that the final sequence corresponds to 4, 5, and 6 fragments. This could indicate that prioritizing the interaction between these sections of the peptide and the CD/ED site and depreciating the rest of the peptide to propose modifications could be an initial approach to find out mimetic peptides.

Despite global scoring values such as MM-PBSA and score only for GNINA not being able to obtain a significant correlation with the peptide’s crystallographic pose on their own, these showed importance when the model decided the RMSD values together with the rest of the variables.

The correlation between the scores obtained in each pose and the similarity to the most realistic pose (those obtained from the crystallographic structure) was also analyzed. The GNINA values for each assembled peptide showed no correlation with the RMSD to the crystallographic pose (*R* = 0.015). Analyzing the more relevant features obtained from the interpretability analysis in each well-fitted model, a formula was inferred with a considerable increase in the correlation between the RMSD and the crystallographic pose (*R* = 0.728).

Thus, a series of rules and an inferred formula (STELLAR score) from these specific rules was conceived. A low value of the STELLAR score implies a more similar pose with respect to the real interaction and pose of the peptide in the CD/ED site of p38alpha. This new metric supposes an interesting approach to rank peptides and other biopolymers that dock in the CD/ED site of p38alpha and mimic the protein–protein interactions that occur with HePTP. This rank could be really useful in order to predict possible allosteric inhibitors from p38α.

By bridging molecular modeling, structural biology, and computational chemistry, STELLAR fosters interdisciplinary collaboration and provides a robust foundation for both academic and industrial applications. Altogether, it represents a transformative step toward more realistic and scalable modeling of flexible biopolymers with far-reaching implications in drug design, precision medicine, and the future of computational biotechnology.

## Limitations

Although the results obtained in this work demonstrate the potential of STELLAR for modeling highly flexible biopolymers, some limitations of the current implementation and validation must be considered.

One of the limitations observed during the initial benchmark execution was the relatively low proportion of systems that could be successfully assembled from the initial dataset (assembly rate). A substantial fraction of these cases required extensive preparation and preprocessing before docking calculations, topology generation, or structural relaxation could be performed. In many instances, issues related to the quality, completeness, or representation of the experimental structures obtained from public databases affected the subsequent reconstruction processes. Consequently, the assembly rate reflects not only the performance of the reconstruction workflow itself but also the challenges associated with the preparation of suitable input structures. Future benchmarking studies would benefit from larger and more carefully curated datasets, although the preparation and validation of such benchmark collections require a considerable manual effort. Nevertheless, the revised pipeline successfully recovered 450 of 500 previously unsuccessful cases, corresponding to a 90% recovery rate.

Another aspect of the current workflow is the use of a short GROMACS relaxation protocol. This stage was designed as a rapid structural refinement step intended to remove steric clashes and correct local geometrical distortions generated during fragment assembly, rather than to reproduce the long time scale conformational dynamics of the complex. The protocol proved to be sufficient for obtaining realistic structures and accurate RMSD calculations within the scope of this work. Nevertheless, future developments could explore longer relaxation procedures or conventional molecular dynamics simulations to further refine the reconstructed complexes.

Beyond improving structural refinement, extended simulations could provide additional dynamic and energetic descriptors that may be incorporated into future ranking strategies, potentially increasing the predictive power and accuracy of the methodology. Such improvements, however, would need to be balanced against the reduction in the high-throughput capabilities that characterize the current workflow.

Regarding the ranking metrics explored in the p38α–HePTP case study, the proposed model should be considered a proof of concept. The metric was derived from different features generated by STELLAR using different assembled poses of the same peptide bound to a single binding site and was specifically designed to prioritize candidate poses within this system. Therefore, the current study demonstrates the feasibility of extracting interpretable ranking metrics from STELLAR-generated descriptors, but does not establish the general applicability of the resulting model to other proteins, binding sites, or ligand families. Additional validation across diverse protein–biopolymer systems will be required to assess the transferability and robustness of this approach.

Although the results obtained for polysaccharide systems were encouraging, the number and diversity of the currently available benchmark cases remain limited. Expanding the evaluation to larger datasets, including branched and structurally heterogeneous polysaccharides, would provide a more comprehensive assessment of the robustness of the workflow across different carbohydrate systems.

The applicability of STELLAR to polysaccharide systems was also evaluated successfully, demonstrating that the workflow can be extended beyond peptides to other classes of flexible biopolymers. Although the results obtained for polysaccharide systems were encouraging, the number and diversity of the currently available benchmark cases remain limited. Expanding the evaluation to larger datasets, including branched and structurally heterogeneous polysaccharides, would provide a more comprehensive assessment of the robustness of the workflow across different carbohydrate systems.

Finally, the primary objective of this study was to demonstrate the applicability of STELLAR for the reconstruction and ranking of highly flexible biopolymers. Because existing docking methods differ substantially in benchmark composition, peptide length restrictions, system preparation protocols, and evaluation criteria, direct comparisons using a common benchmark remain challenging. Future studies based on standardized datasets will be valuable for establishing a more systematic comparison between STELLAR and other available approaches.

## Outlook

STELLAR successfully performs fragment-based docking on peptide–protein benchmarks, achieving RMSD values below 5 Å in most cases. Its applicability to polysaccharide ligands has also been demonstrated. However, future work should explore its performance on other classes of polymers, such as fatty acids, plastic-based molecules, or any polymers with a high number of rotatable bonds that remain challenging for conventional docking methods.

Additionally, a more exhaustive study involving larger polysaccharide–protein complexes, including branched polysaccharides, would further validate the robustness of the method. Although the PDB database contains some polysaccharide–protein complexes suitable for benchmarking, the availability of sufficiently large and diverse cases remains limited. Expanding this dataset would provide a more comprehensive evaluation and reinforce the relevance of STELLAR for complex carbohydrate systems.

One of the main challenges identified during STELLAR benchmarking was the preparation of protein structures, particularly those obtained from the Propedia. Many structures extracted directly from the PDB failed during topology generation or MD simulations because of insufficient preprocessing. Therefore, more rigorous and standardized structure preparation protocols are required to ensure successful MD simulations within the STELLAR pipeline. This improvement would enable the construction of larger and more reliable benchmark datasets. In addition, future developments could explore longer relaxation protocols or conventional molecular dynamics simulations, although these approaches would reduce the high-throughput capabilities of the current workflow.

In this study, we also developed a novel ranking approach for modeling the p38α-HePTP KIM peptide complex. This strategy can be extended to other systems where a known binding site and interacting peptide are available. Furthermore, the use of interpretable machine learning provides valuable insights into the key features governing binding, moving beyond the “black-box” nature of many probabilistic and stochastic approaches.

While a detailed analysis was performed for the p38α case, this methodology can be generalized to other biologically and industrially relevant systems. In particular, it opens new opportunities for identifying large polymer-based candidates as potential hits for experimental validation.

STELLAR currently relies on GNINA as the primary docking engine to generate a broad exploration of the fragment combinations. Future improvements could include the evaluation of alternative parametrizations or the integration of additional docking engines such as Lead Finder or Glide. A consensus scoring strategy combining multiple approaches may further enhance the robustness and reliability of the results.

Finally, although the execution time of STELLAR is competitive with that of existing tools, several bottlenecks remain that could be further optimized. Improvements in MD efficiency, parameter tuning, or the use of faster docking engines could reduce the computational cost, although potential trade-offs with accuracy should be carefully considered.

## Conclusions

The STELLAR protocol represents a significant advancement in peptide–protein docking by enabling accurate and efficient modeling of medium- to large-sized biopolymers (up to 21 subunits). This workflow integrates a fragment-based docking (FDB) strategy using the GNINA engine with additional steps for filtering, assembly, and structural relaxation of the final peptide. The overall pipeline requires minimal GPU resources, achieves competitive execution times, and demonstrates near-linear scalability with respect to the number of fragments. Furthermore, STELLAR leverages the MetaScreener platform to ensure high-throughput performance and reduced computational cost.

The STELLAR workflow was evaluated on a benchmark comprising 265 predictions derived from 257 protein–peptide and 8 protein–polysaccharide complexes. The results consistently produced docking poses with low RMSD values compared to crystallographic structures, confirming its accuracy and robustness across a wide range of ligand sizes and binding environments. The modular design of the protocol, which includes fragment docking, pose recombination, and molecular dynamics refinement, ensures the generation of realistic and stable final conformations.

In addition, a novel scoring function derived from the STELLAR pipeline was successfully applied to the p38α–HePTP KIM peptide complex. This approach showed a strong correlation between the RMSD of the predicted poses relative to the crystallographic structure and the values obtained from the proposed scoring function, demonstrating its potential to rank docking results more reliably than conventional scoring functions. The models and features identified through interpretability analysis also provide a framework for predicting new biopolymer candidates with similar structural characteristics.

Importantly, the interpretability-driven machine learning strategy presented here can be generalized to other systems involving large and flexible biopolymers. By identifying the key features governing binding and structural accuracy, this approach moves beyond traditional “black-box” models and provides mechanistic insight into docking performance.

Overall, STELLAR addresses a critical limitation in current docking methodologies by enabling the scalable and accurate modeling of flexible peptides and polymeric ligands. This protocol establishes a foundation for future applications in the discovery of peptide-based therapeutics and other polymer-derived bioactive compounds.

## Supplementary Material



## Data Availability

A GitHub repository was created to include the version used to launch in the BSC cluster all of the calculations performed in this work. https://github.com/bio-hpc/STELLAR. The final benchmark raw output data contains 265 cases, with an approximate total size of 1 TB. Three cases for each subset (4-fragment peptides, 5-fragment peptides, 6-fragment peptides, 7-fragment peptides, and polysaccharides) have been uploaded to the following Zenodo repository. Input data extracted from Propedia (for peptide–protein complexes) and PDB (for polysaccharide–protein complexes) were also included in the Zenodo link. 10.5281/zenodo.21493247. The remaining data generated by STELLAR is available from the authors upon reasonable request.

## Notes

Workflow descriptions, benchmark analyses, computational performance studies, machine learning evaluation and interpretability analyses, STELLAR score parameters, additional statistical analyses, assembly-failure analysis, and p38α–HePTP interaction studies (PDF)
