# Supplementary material for: STELLAR: A Fragment-Based Docking Workflow for Highly Flexible Long-Chain Biopolymers
Source: J Chem Inf Model. 2026 Sep 16;66(19):12645–66. doi: 10.1021/acs.jcim.6c00987 (PMC13663479; doi:10.1021/acs.jcim.6c00987)
Supplement: Supplementary file 1 [file ci6c00987_si_001.pdf]

Supplementary information

STELLAR: A Fragment-Based Docking Workflow for Highly Flexible Long-Chain Biopolymers

Alejandro Rodríguez-Martínez[1,2], Jochem Nelen[1], Miguel Carmena-Bargueño[1], Carlos Martínez-Cortés[1], Horacio Pérez-Sánchez[1,*]

[1]Structural Bioinformatics and High Performance Computing Research Group (BIO-HPC), UCAM Universidad Católica de Murcia (UCAM), HiTech Innovation Hub, Murcia, 30107, Spain

[2]Health Sciences PhD Program, Universidad Católica de Murcia UCAM, Campus de los Jerónimos nº135, Guadalupe, Murcia, 30107, Spain

* Corresponding Author. Email: hperez@ucam.edu

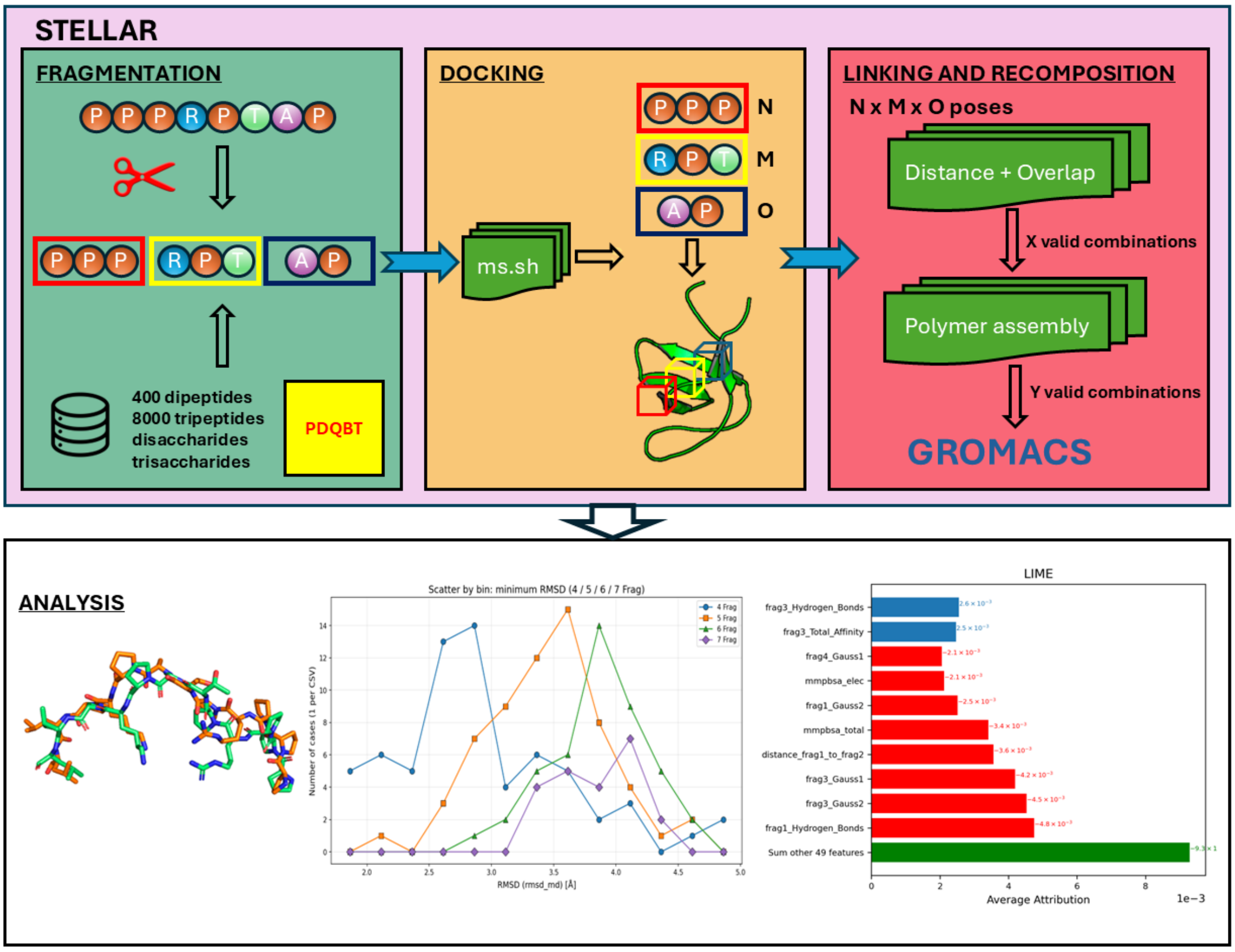

Figure S1. Stepwise overview of the STELLAR workflow. First, the target polymer is fragmented and the specific fragments required for each case are obtained. Subsequently, the selected fragments are docked using predefined coordinates for each system. The combinations of poses obtained from the docking calculations are filtered to retain only valid assemblies based on bond distance and steric overlap thresholds. Finally, fragment reassembly is performed using RDKit and GROMACS, allowing relaxation of the final pose. The resulting structures are analyzed in a post-processing step using different tools to compare RMSD values with the crystallographic pose, evaluate the applied benchmark, and generate a machine learning model for one specific case, including analysis of its machine learning interpretability.

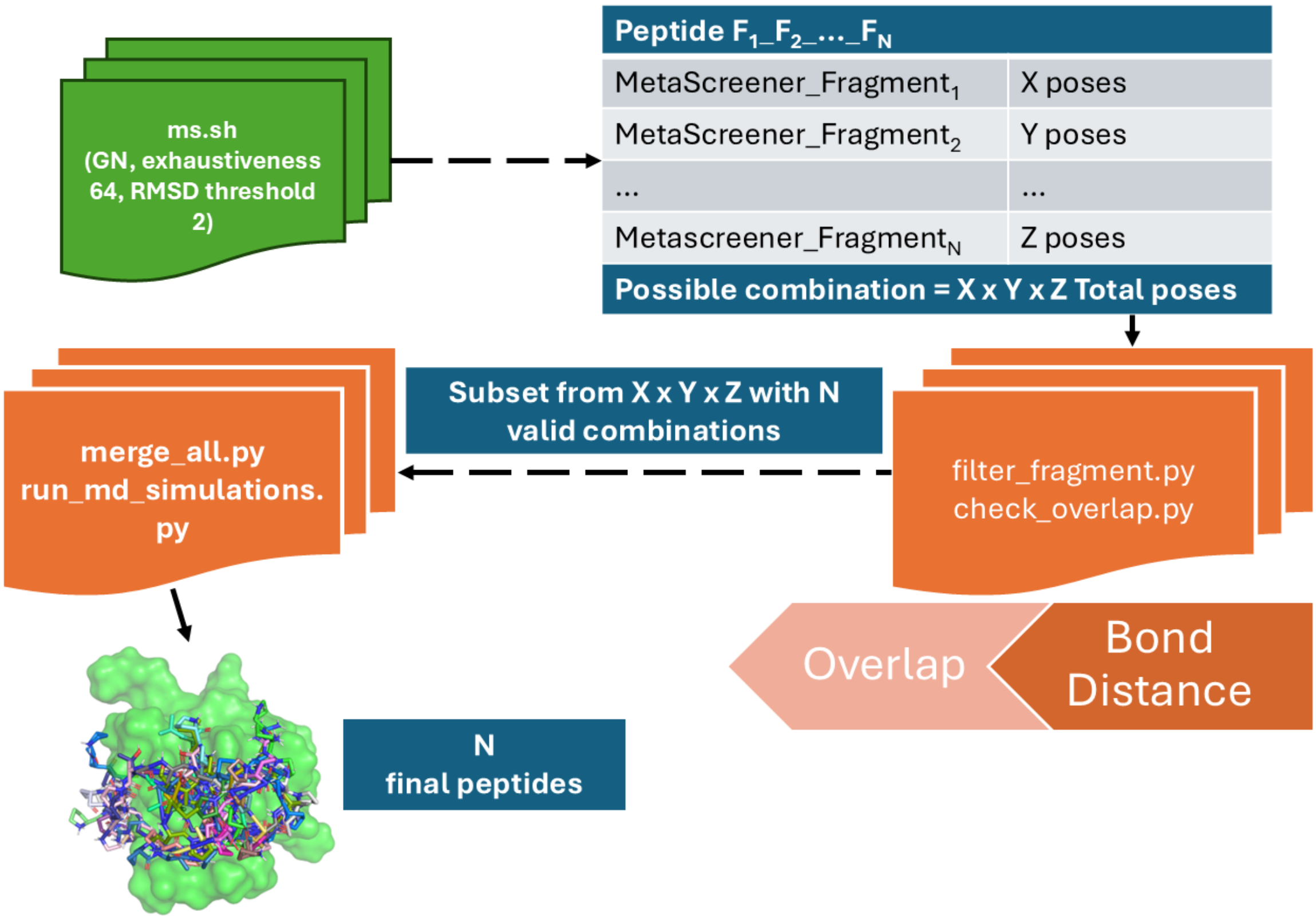

Figure S2. Workflow for selecting and joining valid peptide fragment poses. Each fragment (F_1 to F_n) may generate multiple docking poses via MetaScreener using GNINA software. The script check_fragments.py filters combinations based on sequence order, peptide bond geometry, and atomic overlap. Validated combinations are then passed to join_fragments.py, which fuses fragments into full-length peptides. This process reduces a combinatorially large pool of fragment poses (X × Y × Z) to a final set of N structurally valid, linked peptides.

Algorithm 1 Peptide Pose Filtering and Validation

Require: Fragments $F_1, F_2, \ldots, F_N$, Poses $P_{F_1}, P_{F_2}, \ldots, P_{F_N}$
Ensure: Valid Poses combinations

Step 1: Filter by Peptide Bond Distance
Initialize $Poses_{FilterStep1} \leftarrow \emptyset$
for all pose pairs (i, j) in $Poses_{FilterStep1}$ **do**
    if $distance(coordC_{P_{F_i}}, coordN_{P_{F_j}}) < PeptideBondDistance$ **then**
        Add (P_{F_i}, P_{F_j}) to $Poses_{FilterStep1}$
    end if
end for
Step 2: Filter by Overlap
Initialize $Poses_{FilterStep2} \leftarrow \emptyset$
for all pose pairs (i, j) in all poses **do**
    if $Volume_{P_{F_i}} + Volume_{P_{F_j}} < Volume_{P_{F_i} P_{F_j}} + Tolerance$ **then**
        Add (P_{F_i}, P_{F_j}) to $Poses_{FilterStep2}$
    end if
end for
Step 3: Output Valid Combinations
for all combinations $(P^i_{F_1}, P^i_{F_2}, \ldots, P^i_{F_N})$ in $Poses_{FilterStep3/4}$ **do**
    Copy $P^i_{F_1}, P^i_{F_2}, \ldots, P^i_{F_N}$ to $Peptide_i$
    Print `"Valid peptide structure:` $Peptide_i$`"`
end for

Figure S3. STELLAR algorithm for peptide–protein docking. The protocol comprises multiple filtering steps to identify valid fragment combinations, based on peptide bond distance and overlap between adjacent poses. The selected combinations are subsequently merged during the final join step to generate complete docking solutions.

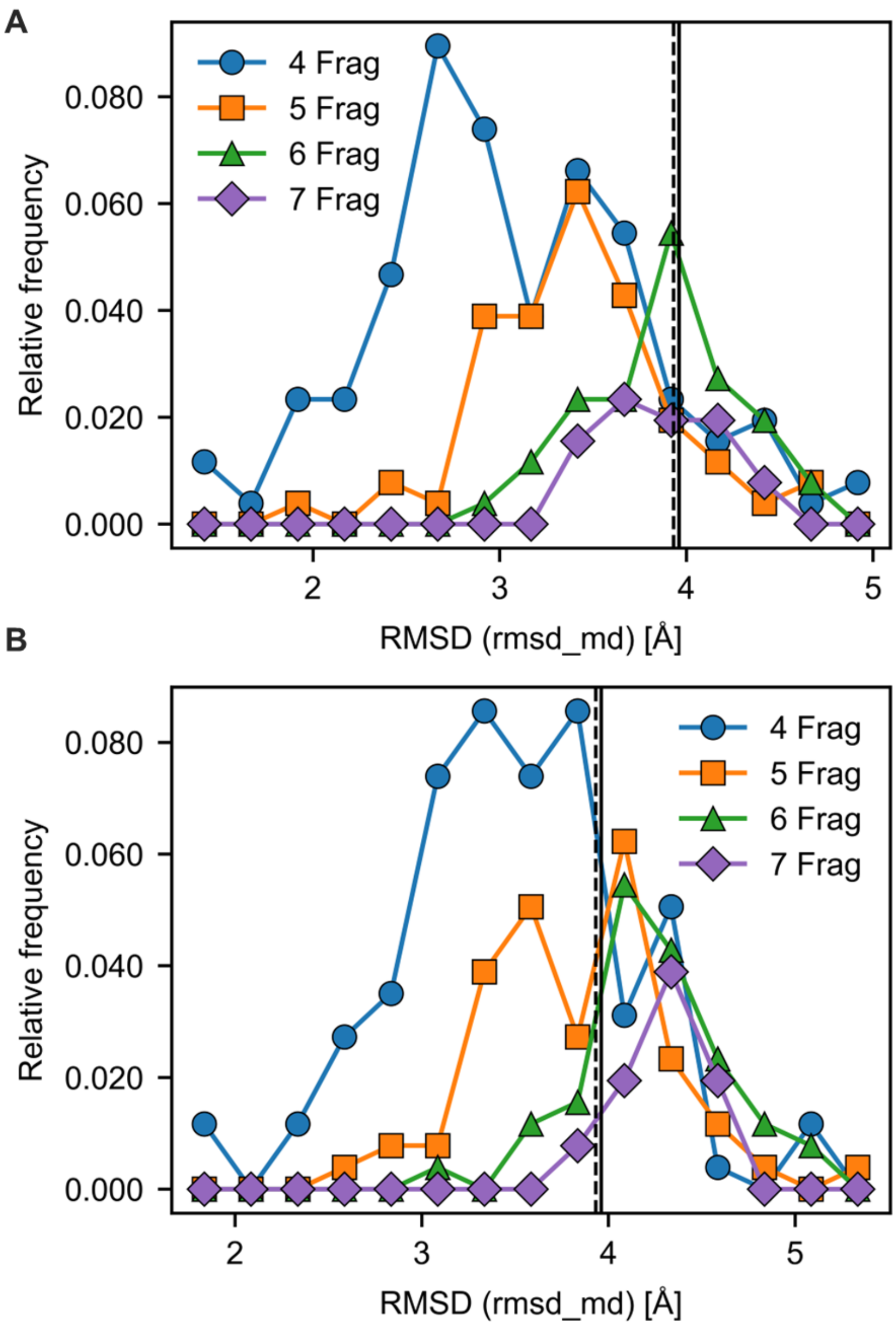

Figure S4. Distribution of minimum RMSD (A) and mean RMSD (B) per case for final assembled peptides with 4, 5, 6, and 7 fragments. The y-axis shows relative frequency with respect to the full set of cases. The solid black line indicates the median RMSD, while the dashed black line indicates the mean RMSD, both calculated from all individual RMSD_md values across the subsets.

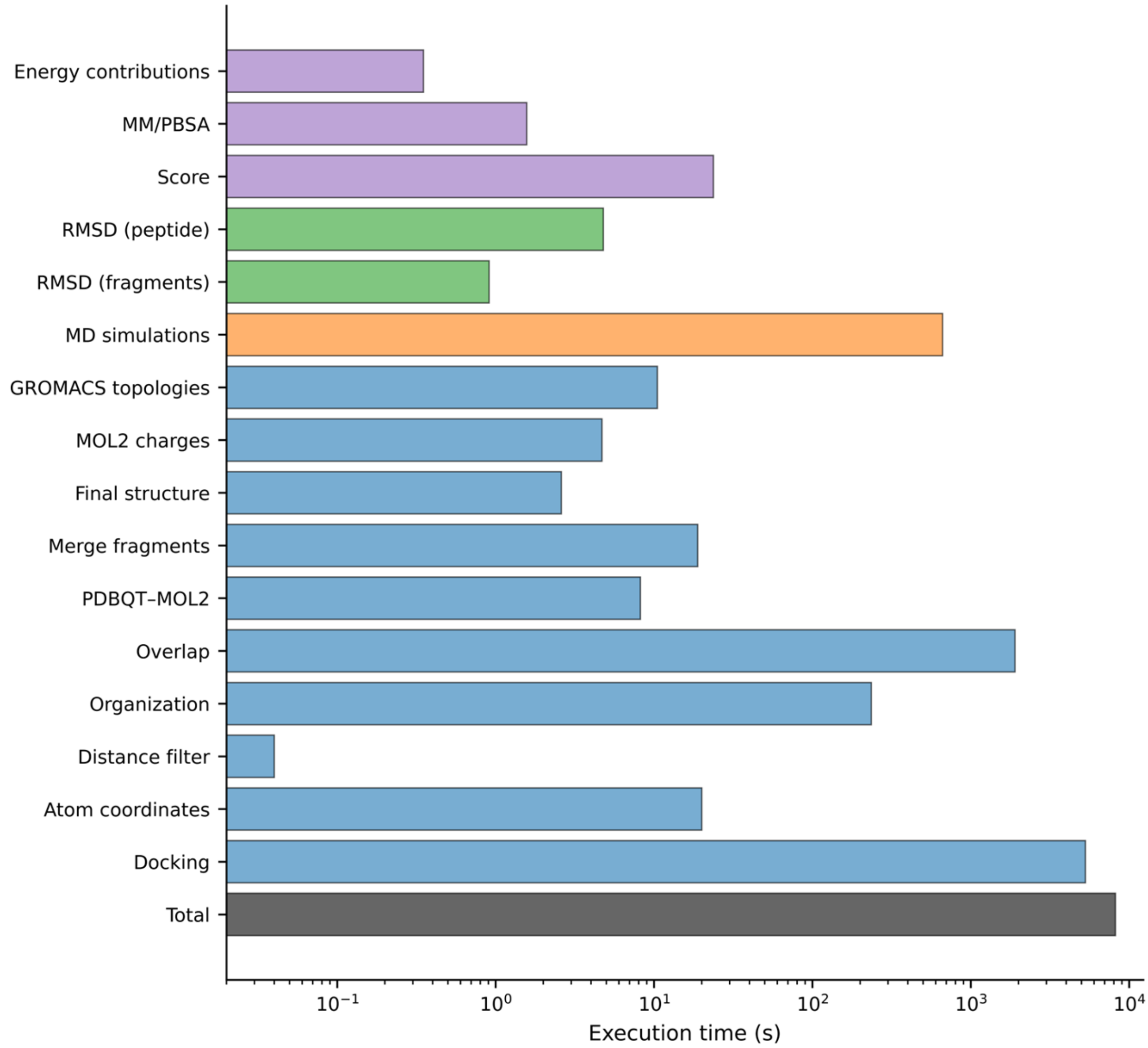

Figure S5. Execution time for a single STELLAR run (one processed combination) on the protein–peptide complex from PDB entry 3R7G (7 fragments). The x-axis shows the execution time in seconds on a logarithmic scale, while the y-axis represents the different steps of the STELLAR workflow. The execution time is divided into four main stages: docking, filtering, and joining (blue); relaxation via molecular dynamics (MD) simulation (orange); structural analysis through RMSD calculations relative to the crystallographic pose (green); and extraction and calculation of additional energy metrics (purple). The total execution time (black) for a single combination in the 3R7G case (7 fragments) was 8,187.49 seconds (2.27 hours).

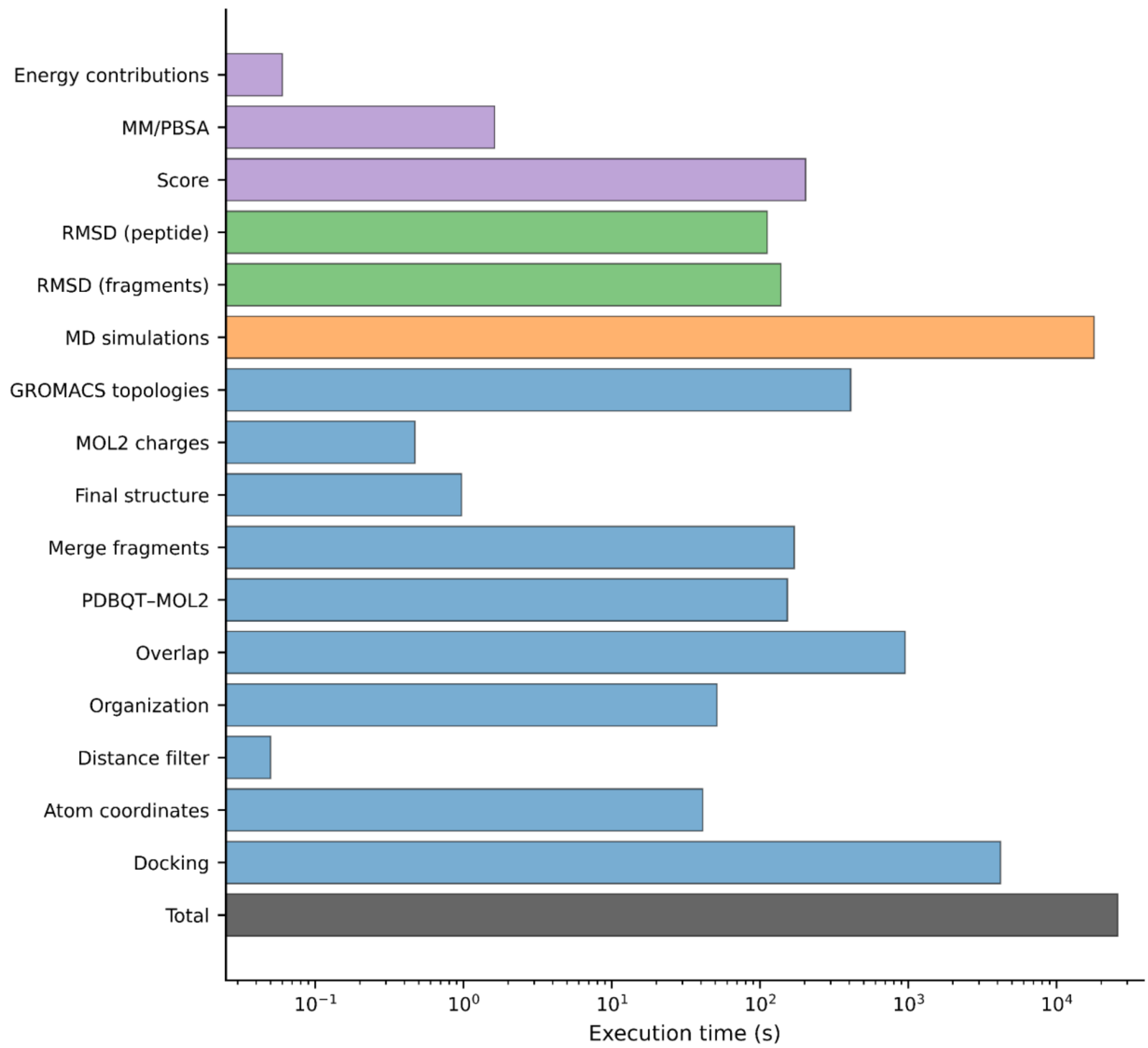

Figure S6. Cumulative execution time for a complete run of STELLAR for 100 combinations in the protein-peptide from 2ND1 PDB (5 fragments). The execution time was divided into four steps: docking, filtering, and joining (blue); relaxation via MD simulation (orange); structural analysis through RMSD calculations against the crystallographic pose (green); and extraction and calculation of additional energy metrics (purple). The total execution time (black) for a single combination in the 2ND1 case (5 fragments), with 100 combinations processed, was 25846.22 seconds (7.18 hours).

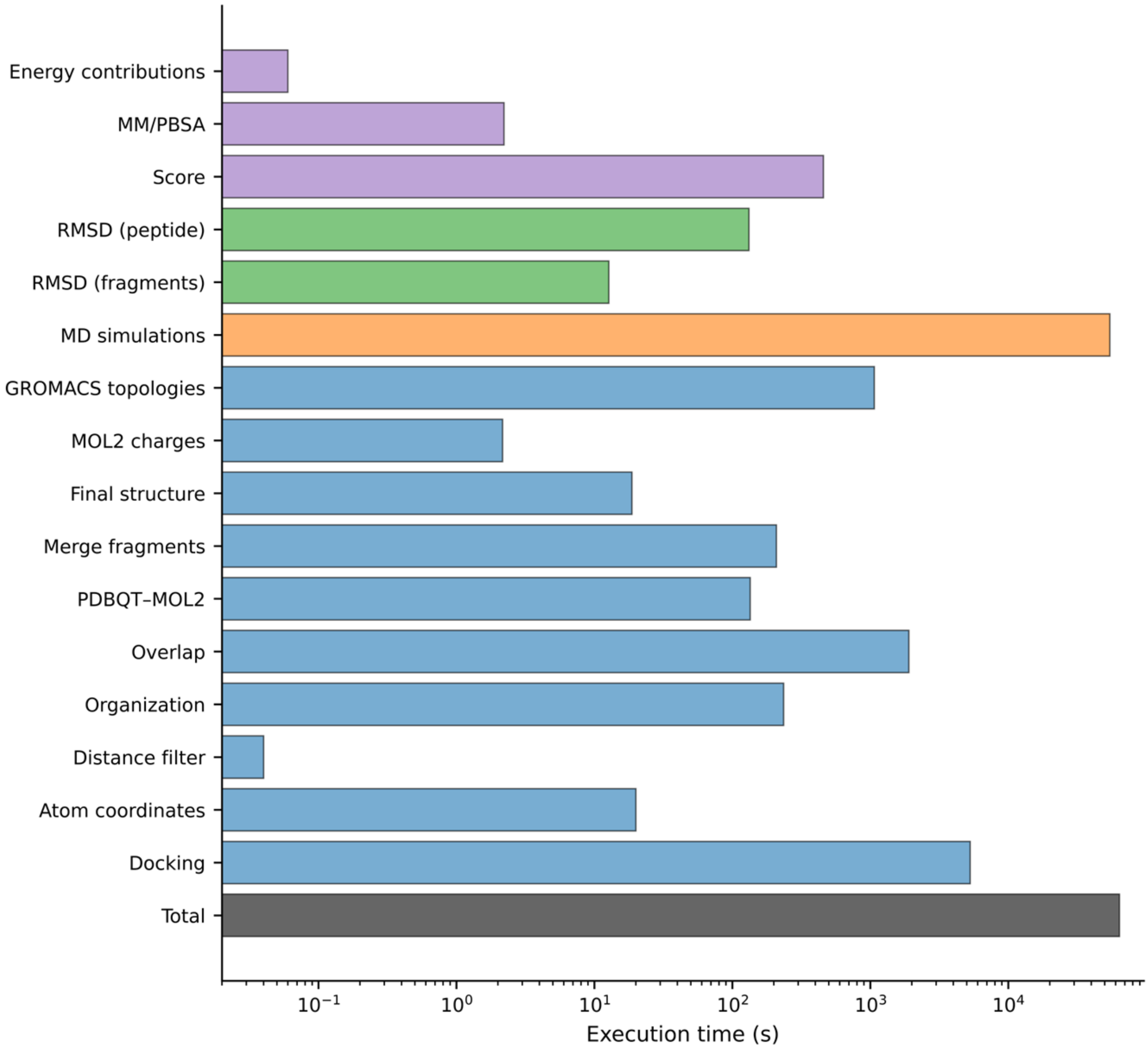

Figure S7. Cumulative execution time for a complete run of STELLAR for 100 combinations in the protein-peptide from 3R7G PDB (7 fragments). The execution time was divided into four steps: docking, filtering, and joining (blue); relaxation via MD simulation (orange); structural analysis through RMSD calculations against the crystallographic pose (green); and extraction and calculation of additional energy metrics (purple). The total execution time (black) for a single combination in the 3R7G case (7 fragments), with 100 combinations processed, was 64027.45 seconds (17.79 hours).

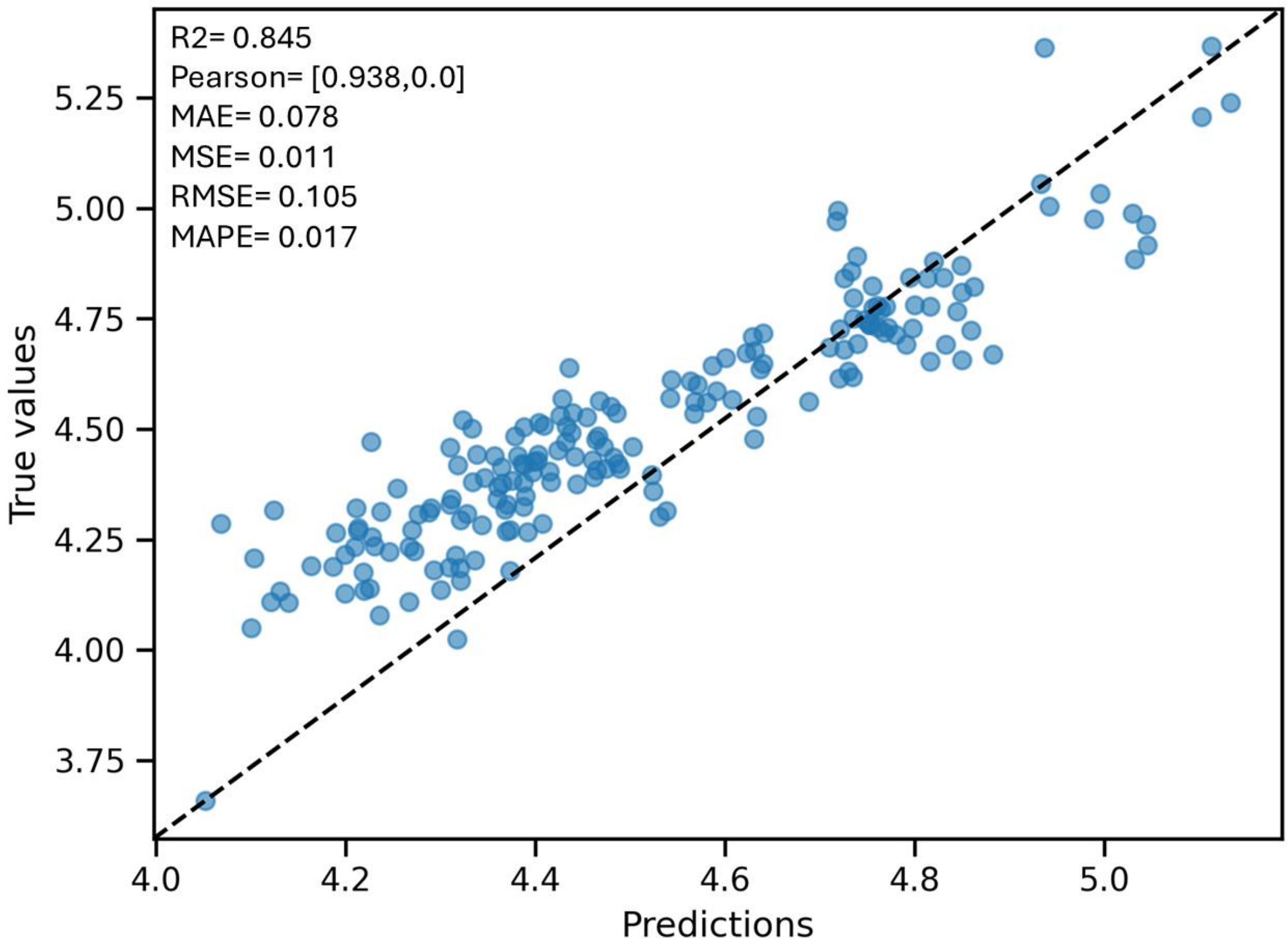

Figure S8. Scatter plot of predicted versus true values for the Bootstrap Aggregating model (R^2 = 0.845).

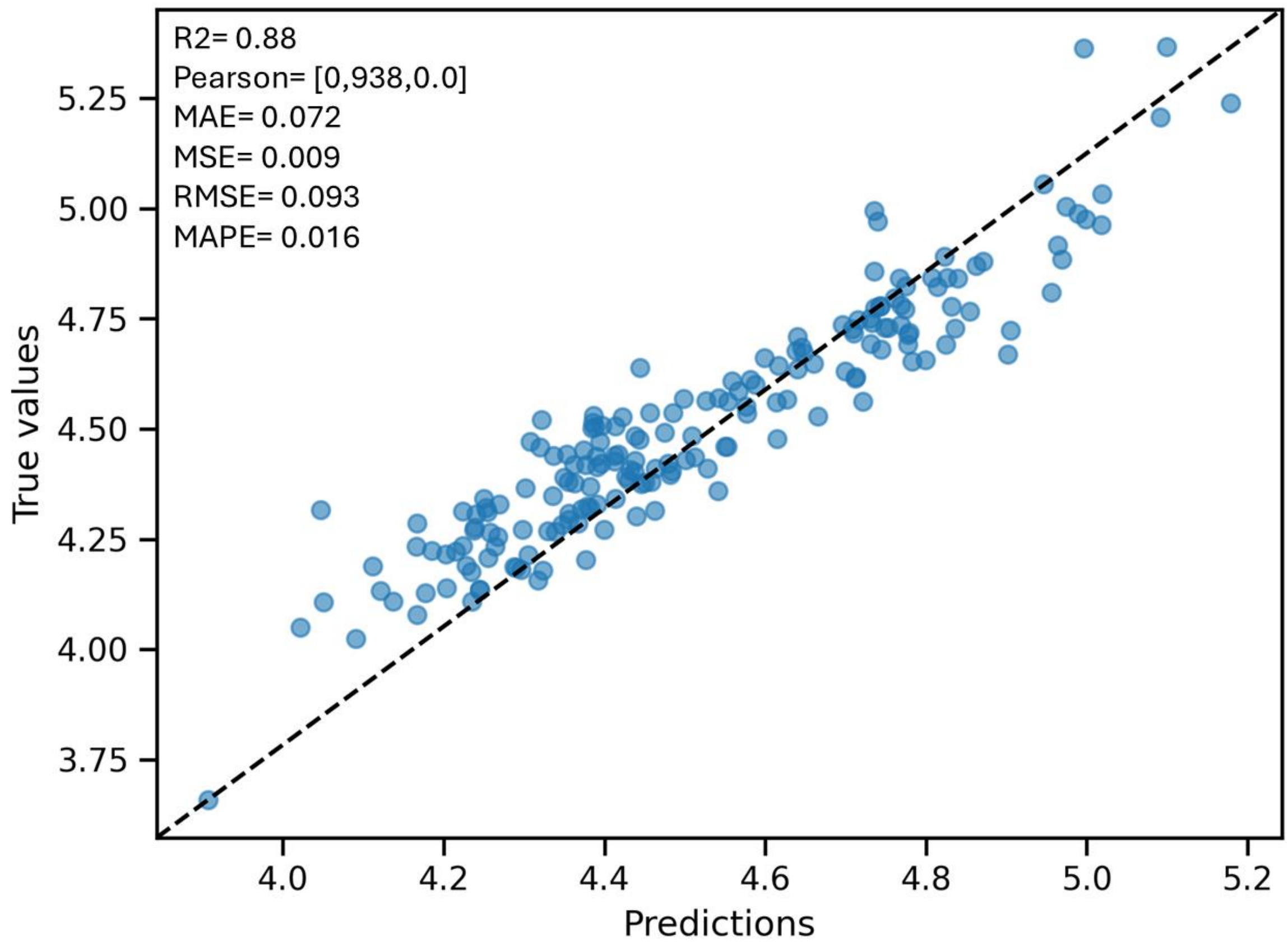

Figure S9. Scatter plot of predicted versus true values for the Random Forest model (R^2 = 0.88).

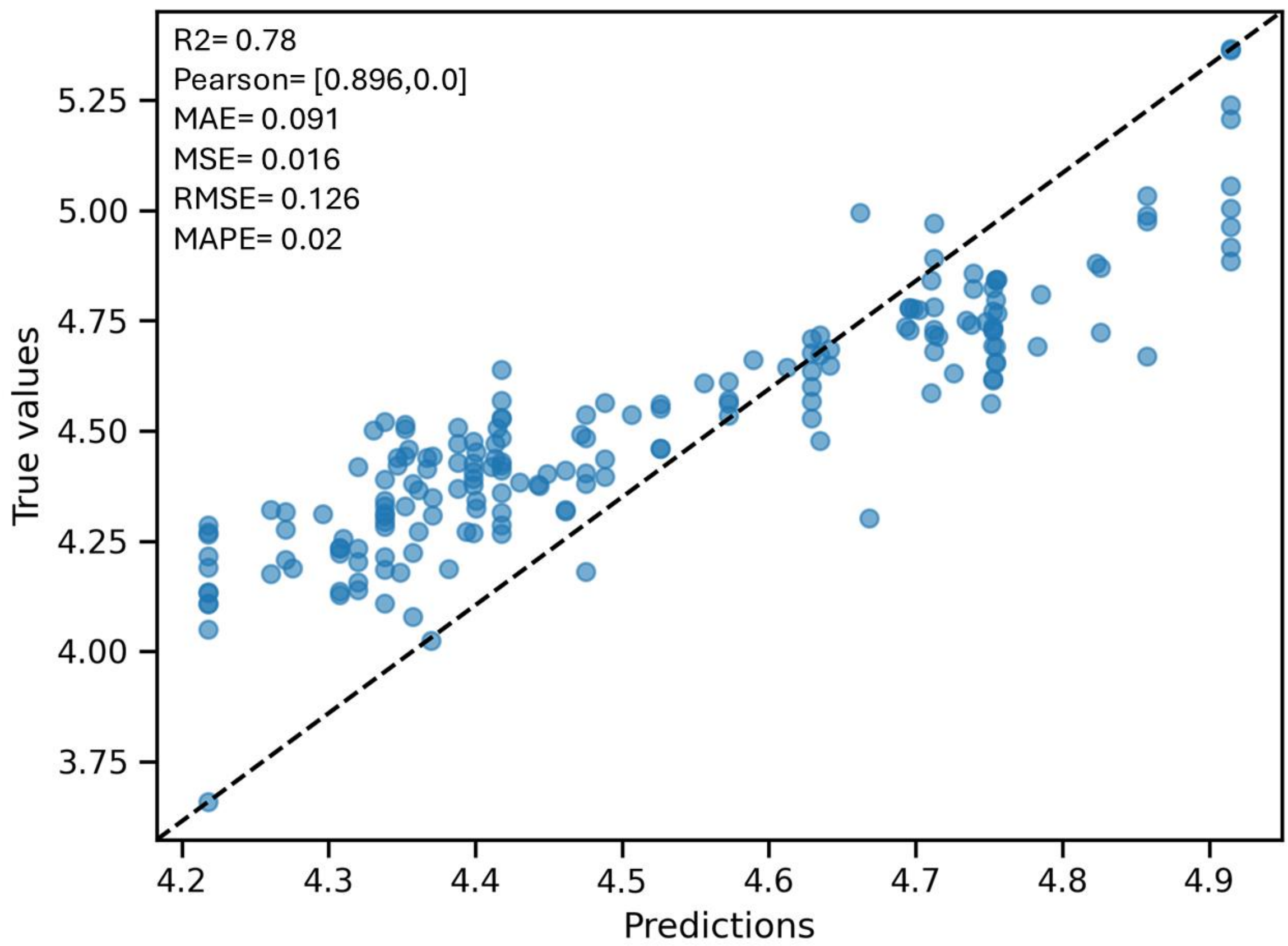

Figure S10. Scatter plot of predicted versus true values for the XGBoost model (R^2 = 0.78).

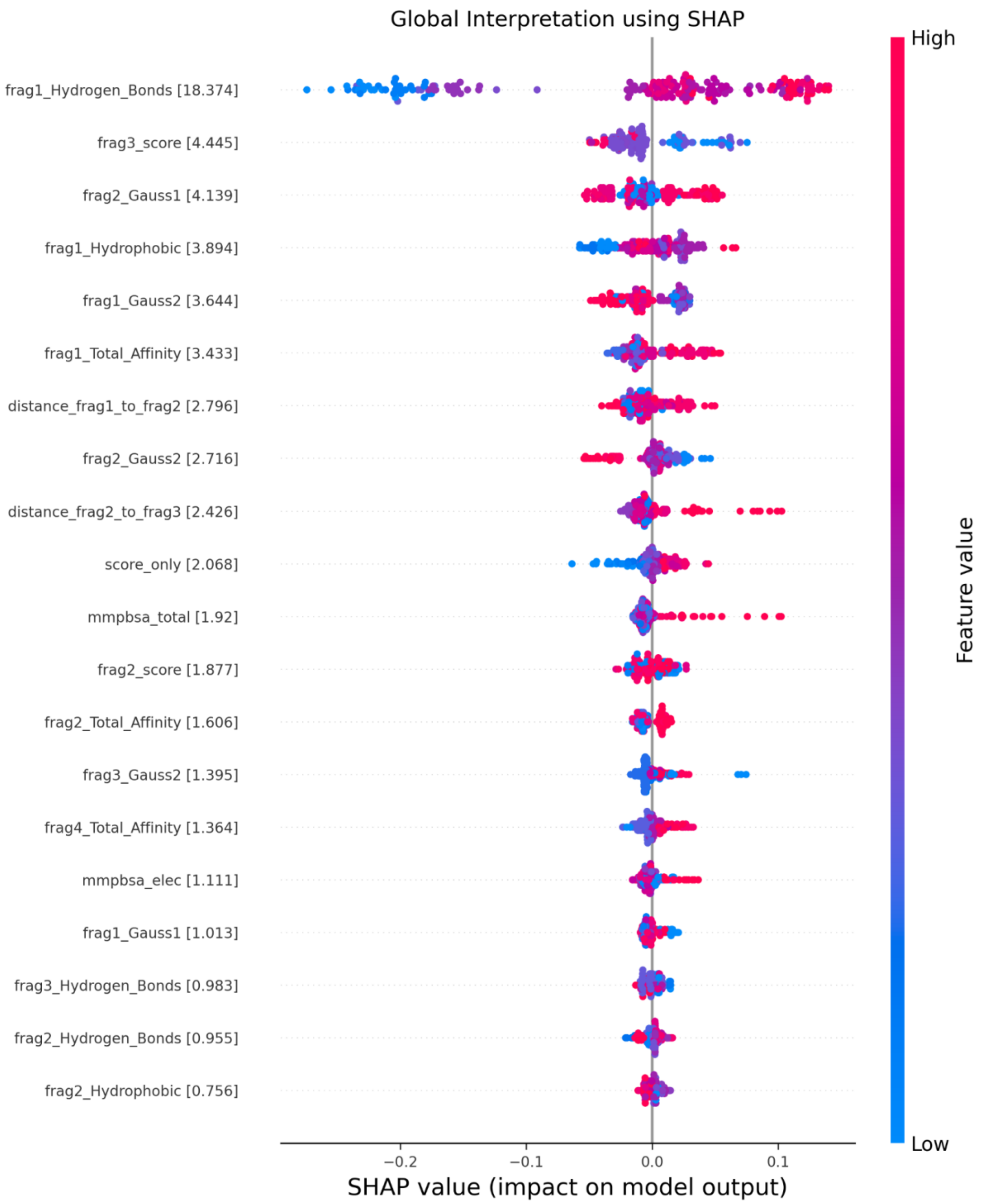

Figure S11. Shapley Additive Explanations (SHAP) plot for the Bootstrap Aggregating model, showing feature-wise SHAP value distributions and feature-value color coding.

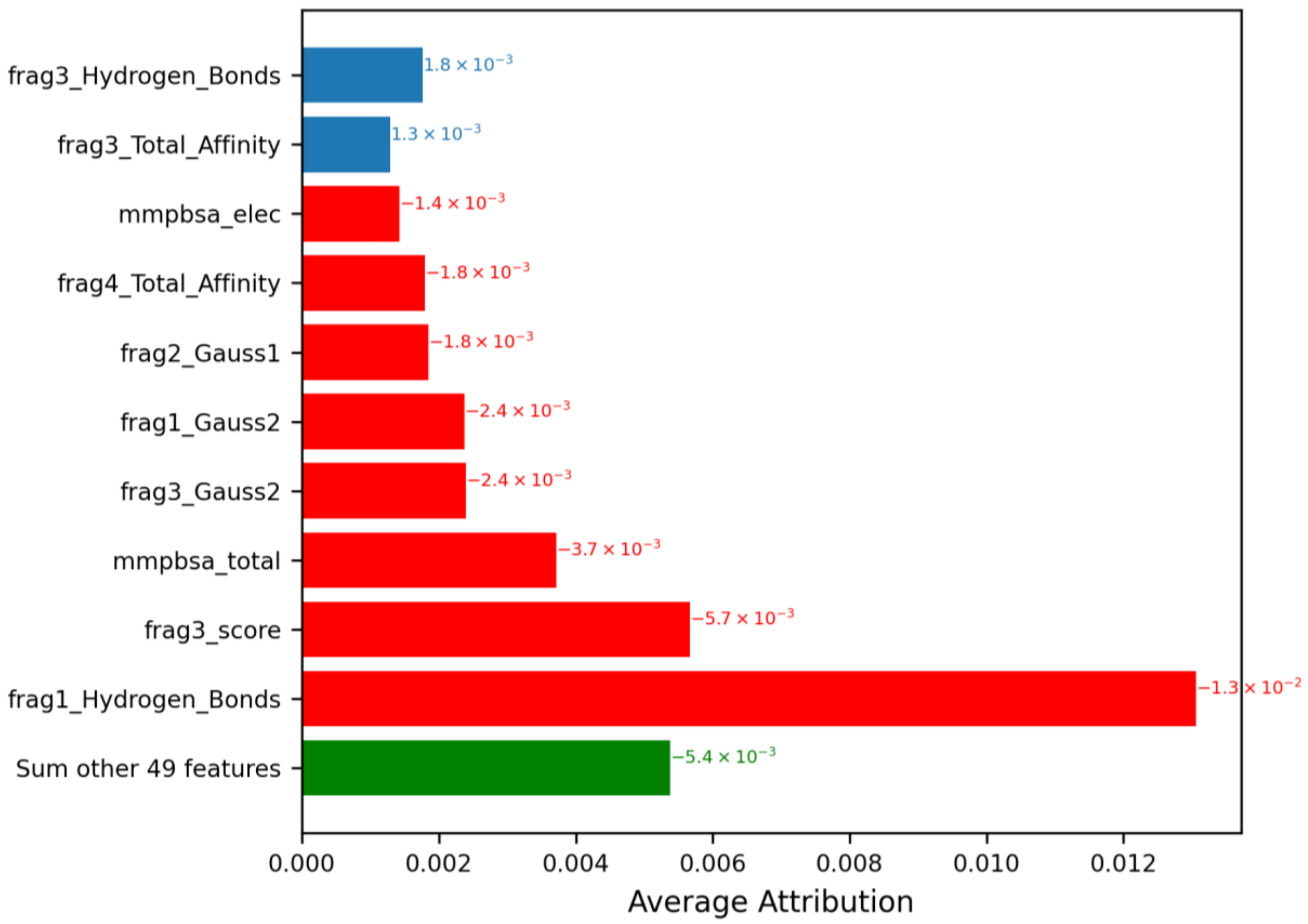

Figure S12. LIME bar chart for the Bootstrap Aggregating model. The plot shows the average local feature attributions, including the sum of the remaining features' contributions.

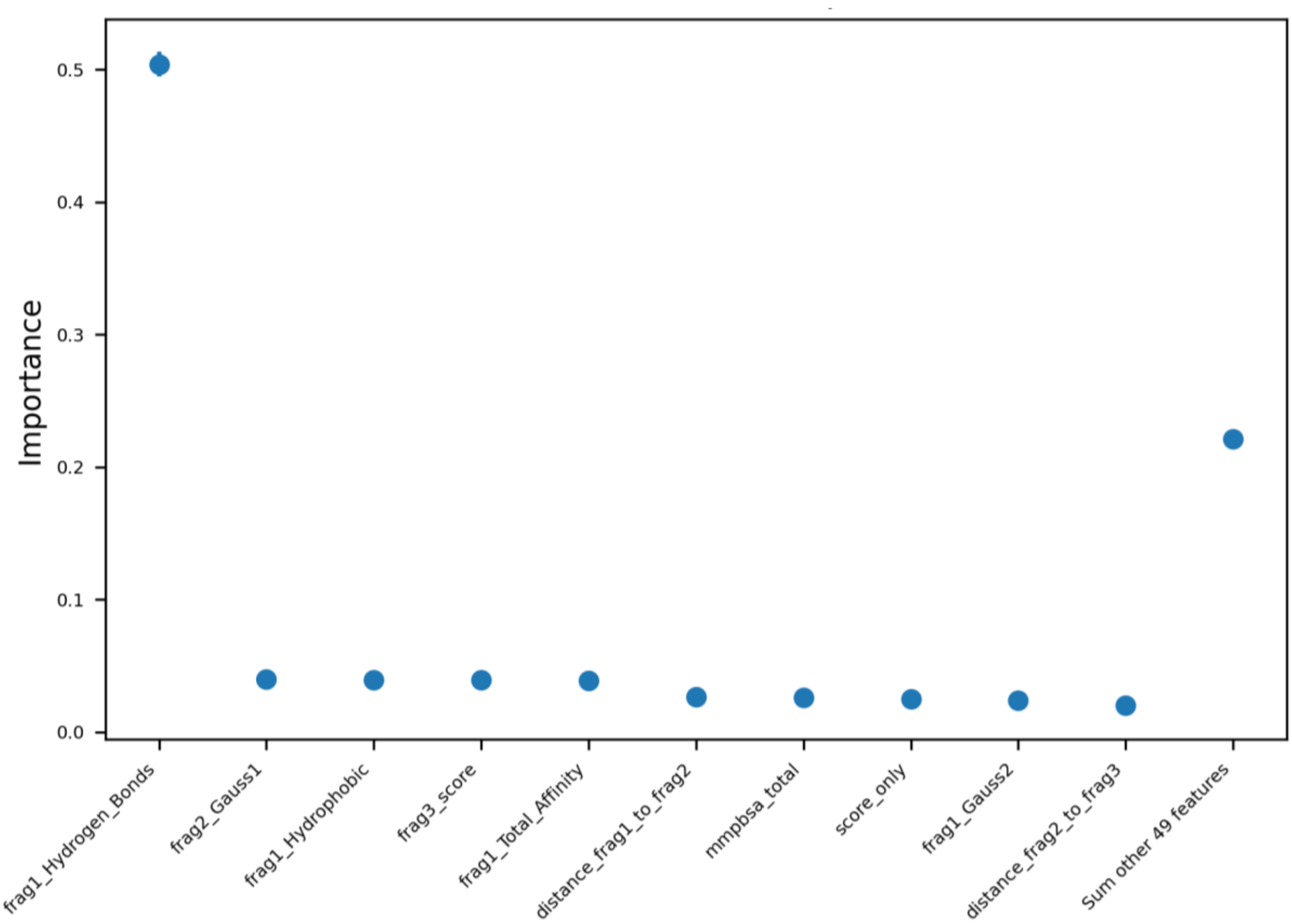

Figure S13. Permutation feature importance for the Bootstrap Aggregating model. The plot shows the contributions of the 10 most important predictors, along with the sum of the remaining features' contributions, expressed as average attribution.

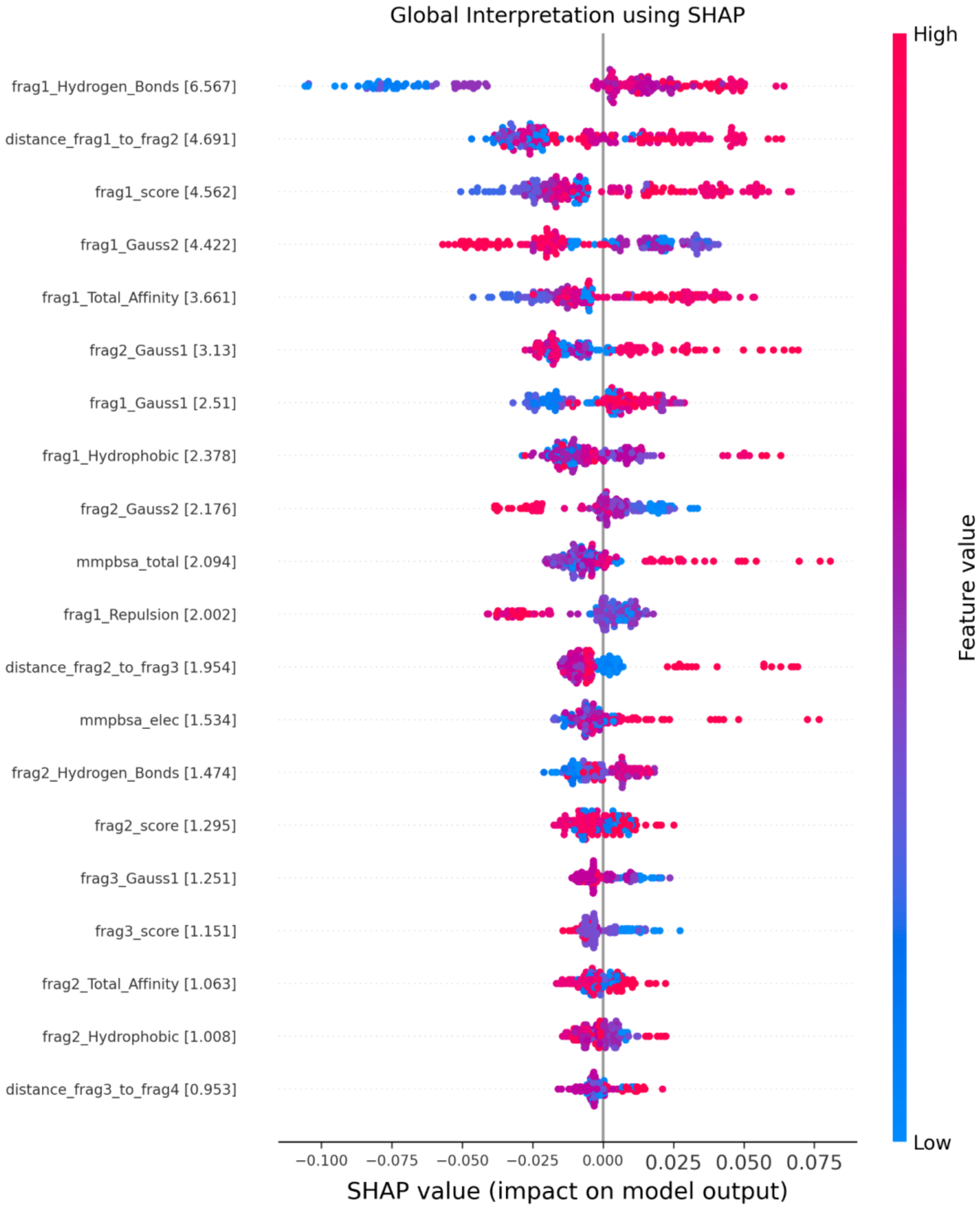

Figure S14. Shapley Additive Explanations (SHAP) plot for the Random Forest model, showing feature-wise SHAP value distributions and feature-value color coding.

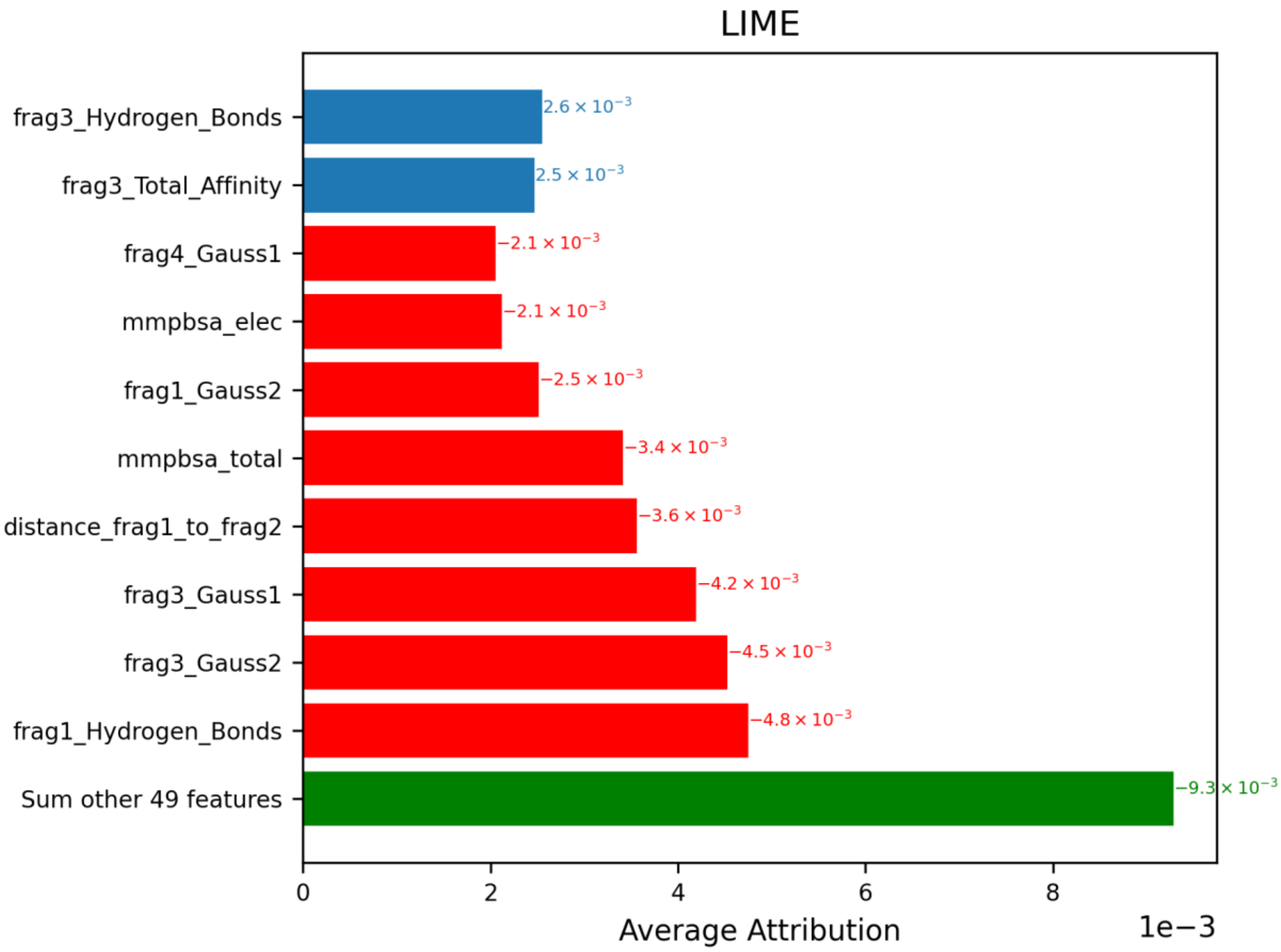

Figure S15. LIME bar chart for the Random Forest model. The plot shows the average local feature attributions, including the sum of the remaining features' contributions.

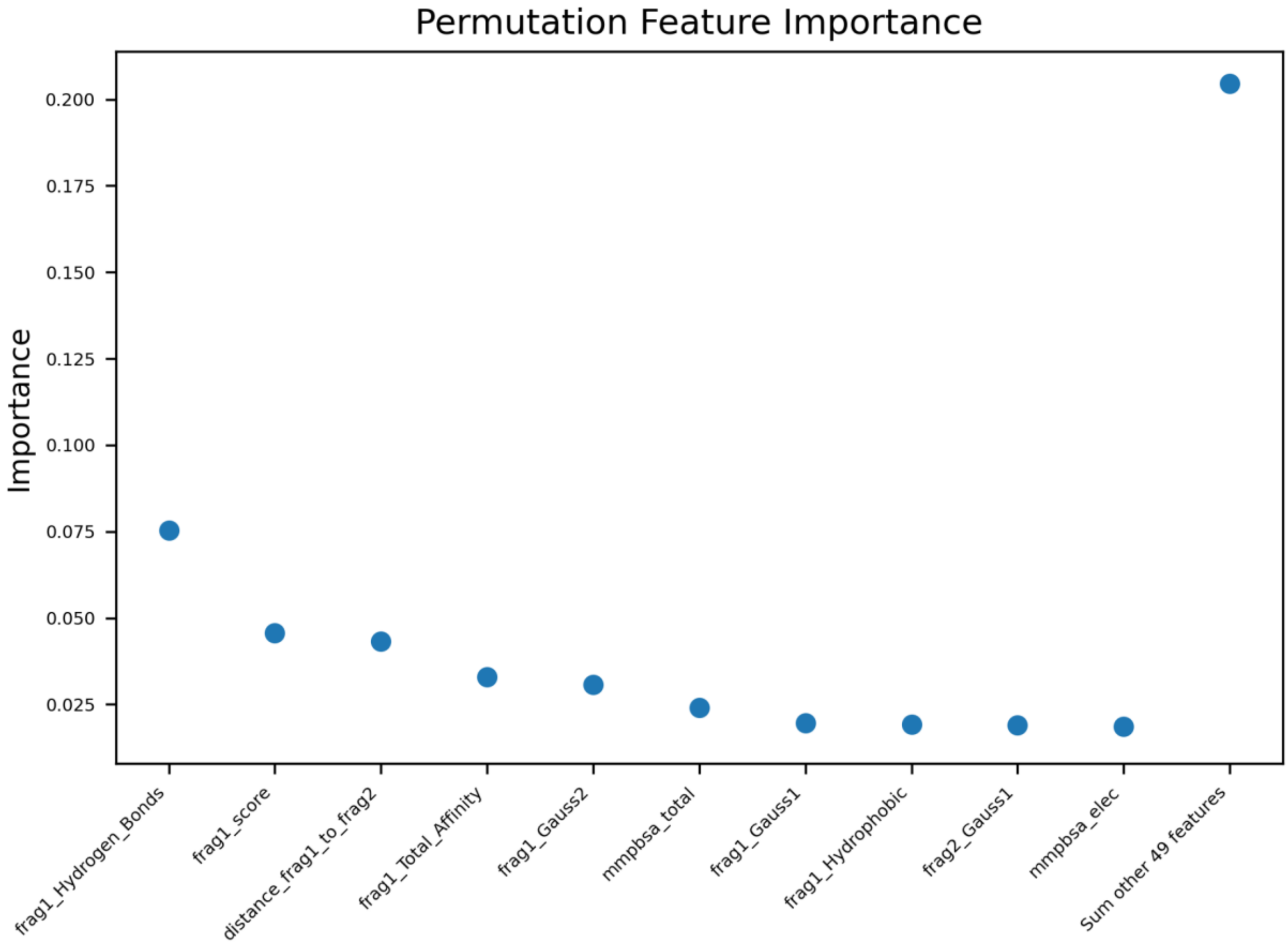

Figure S16. Permutation feature importance for the Random Forest model. The plot shows the contributions of the 10 most important predictors, along with the sum of the remaining features' contributions, expressed as average attribution.

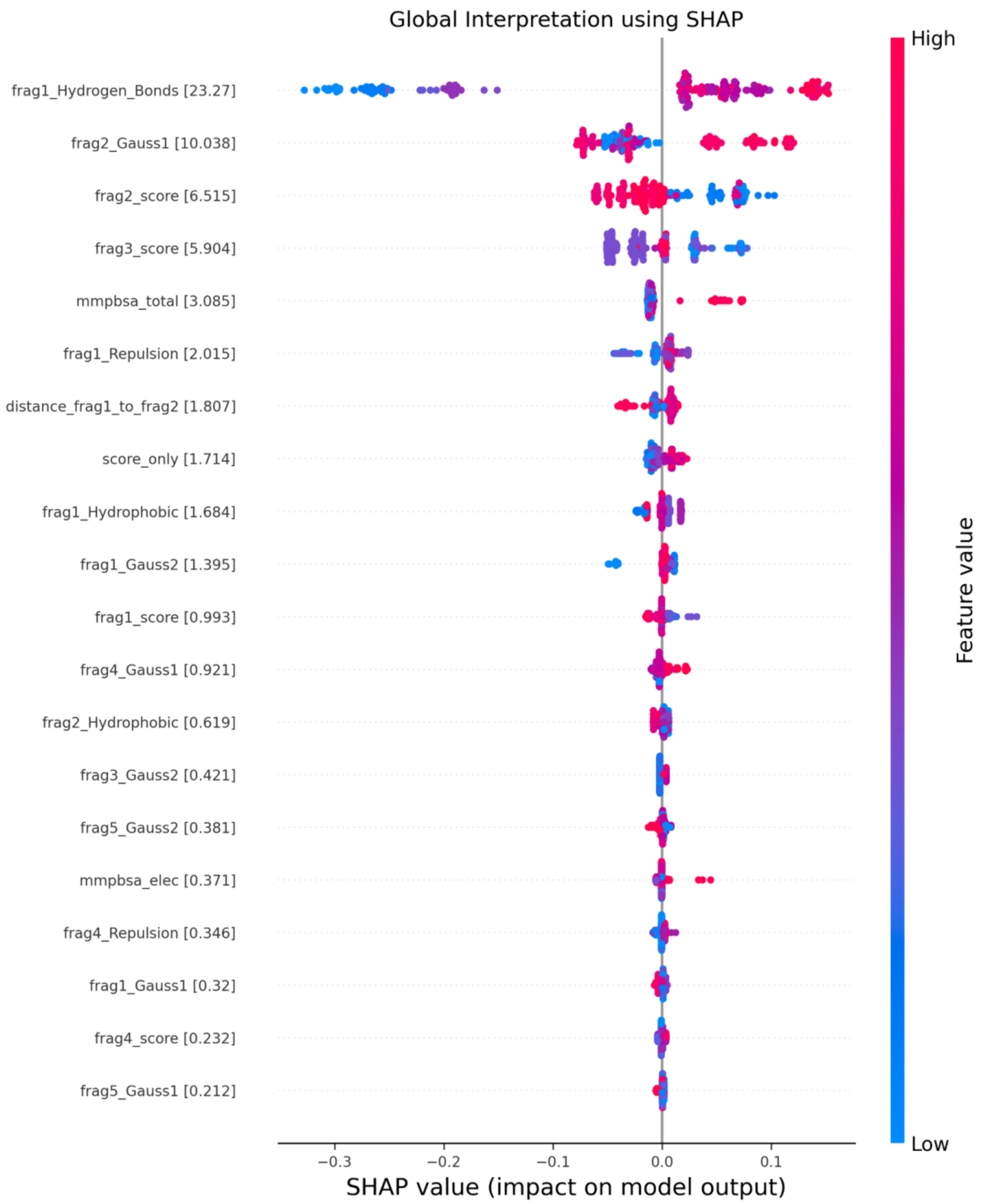

Figure S17 Shapley Additive Explanations (SHAP) plot for the XGBoost model, showing feature-wise SHAP value distributions and feature-value color coding.

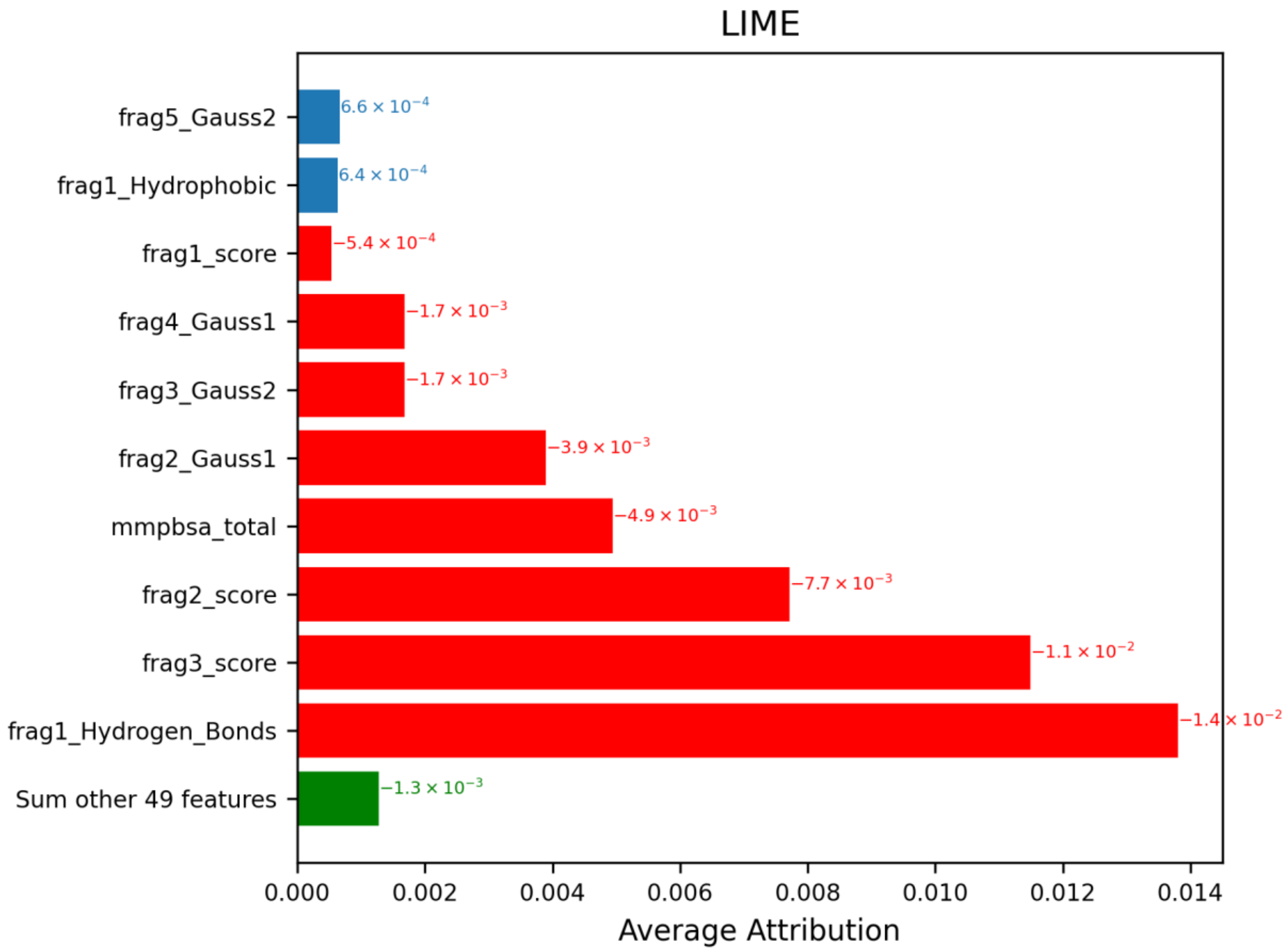

Figure S18 LIME bar chart for the XGBoost model. The plot shows the average local feature attributions, including the sum of the remaining features' contributions.

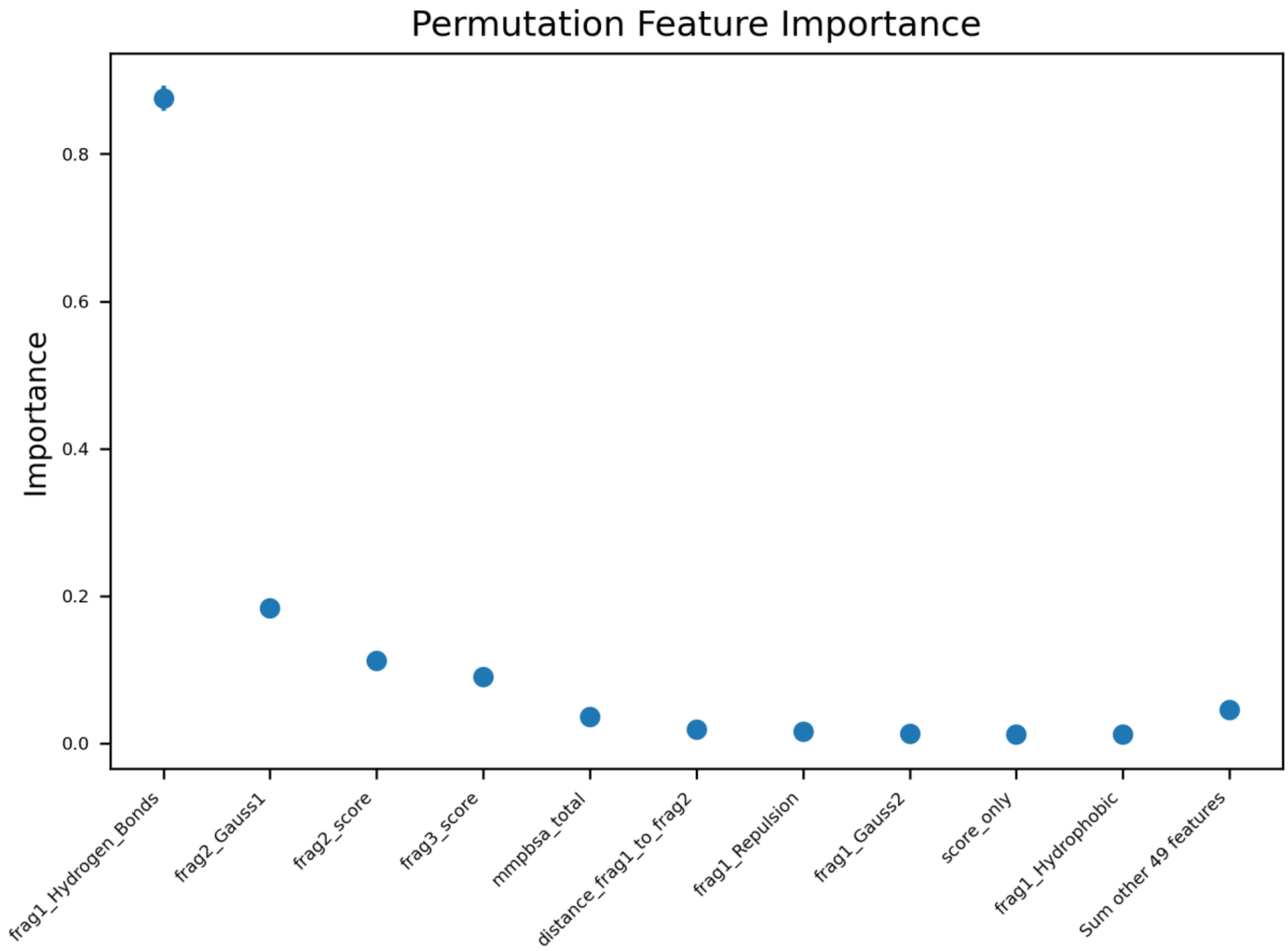

Figure S19. Permutation feature importance for the XGBoost model. The plot shows the contributions of the 10 most important predictors, along with the sum of the remaining features' contributions, expressed as average attribution.

A

		HePTP KIM peptide		
		R16	L17	Q18
P38α CT/ED site	TYR132	■		
	TYR311	■		
	ARG136	■		
	ASP161			■
	GLU163			■

B

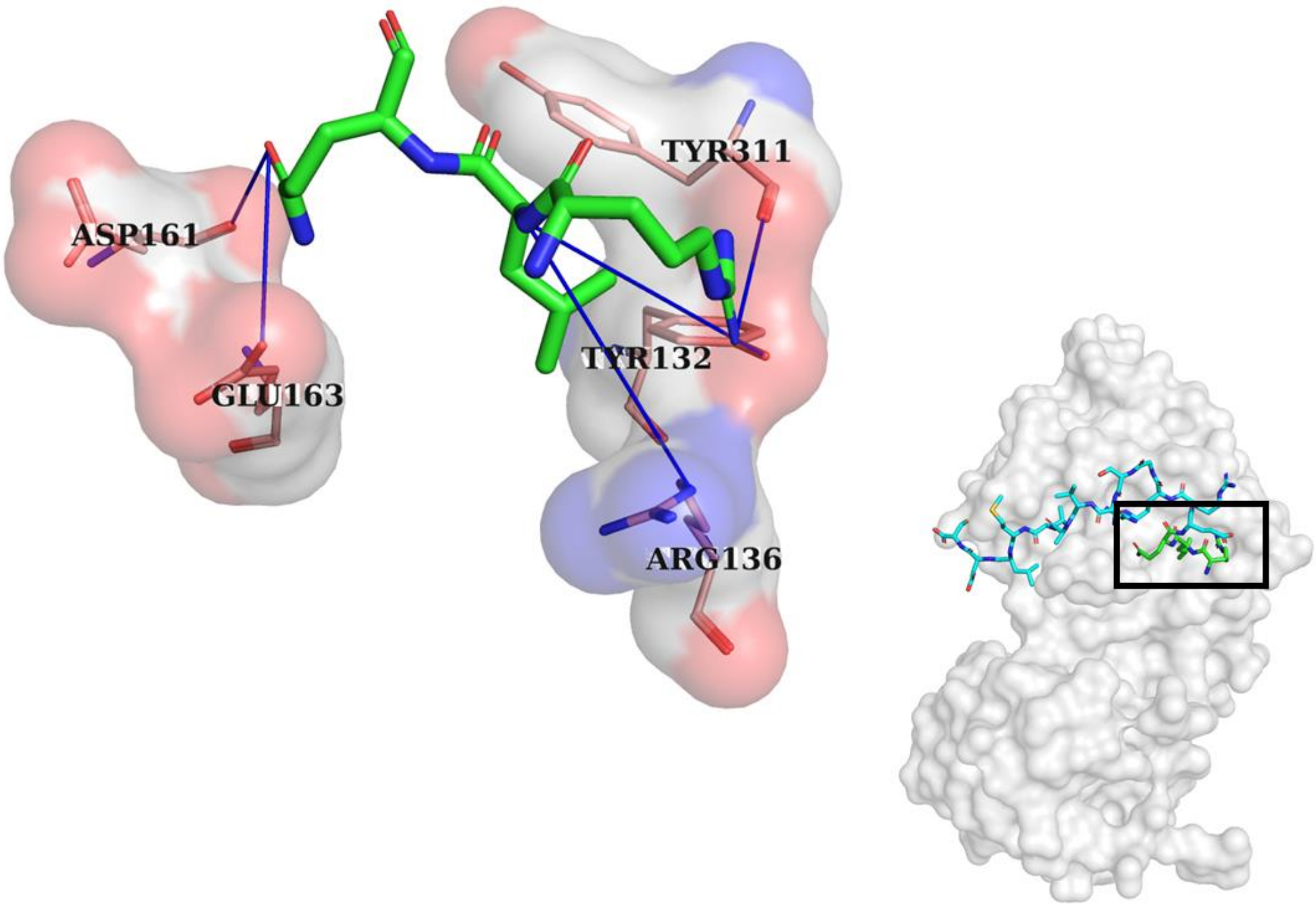

Figure S20. (A) Interaction matrix showing hydrogen bonds between key residues of the p38α CT/ED docking site and the HePTP KIM peptide (residues R16–Q18). (B) Structural representation of the HePTP KIM peptide bound to the p38α CT/ED site from the crystal structure (PDB ID: 8YP8). Hydrogen bond interactions identified by PLIP are shown as dashed lines. The electrostatic surface of p38α is displayed to highlight the interaction environment.

Table S1. Model coefficients for each descriptor included in the STELLAR score for the p38α D-recruitment site binding case. The model comprises the intercept term plus the 15 descriptors (16 linear coefficients in total), together with 5 pairwise interaction terms. Thirteen descriptors were used without transformation, whereas three were transformed: inverse distance was applied to *distance_frag1_to_frag2* and *distance_frag2_to_frag3*, and a logarithmic transformation was applied to *mmpbsa_elec_raw*. Five interaction terms were additionally included.

Descriptor	Coefficient
Intercept	31.449
frag1_Hydrogen_Bonds	+1.157
frag3_score	−26.581
frag2_Gauss1	+0.034
mmpbsa_total	+0.019
frag1_Gauss2	+5.711
frag1_Total_Affinity	+6.918
frag1_Hydrophobic	+0.395
frag3_Gauss2	−30.920
frag4_Total_Affinity	+0.195
log(-mmpbsa_elec_raw)	−9.633
frag2_Gauss2	−0.554
frag3_Hydrogen_Bonds	−0.042
score_only	+0.134
inv_distance_frag1_to_frag2	+0.181
inv_distance_frag2_to_frag3	+0.444
frag1_Hydrogen_Bonds × frag1_Total_Affinity	+0.206
frag1_Gauss2 × frag1_Total_Affinity	+1.243
frag3_score × frag3_Hydrogen_Bonds	+0.144
frag3_score × frag3_Gauss2	−10.851
log(-mmpbsa_elec_raw) × mmpbsa_total	−0.003

Table S2. Success ratio for each benchmark subset evaluated with STELLAR. A prediction is considered successful when the RMSD (minimum or mean across combinations) is below 5 Å.

Subset	Min RMSD		Mean RMSD	
	Success Cases/ Total	Success ratio (%)	Success Cases /Total	Success ratio (%)
4 Fragment-peptides	129 / 129	100.00	126 / 129	97.67
5 Fragment-peptides	62 / 62	100.00	61 / 62	98.38
6 Fragment-peptides	44 / 44	100.00	42 / 44	95.45
7 Fragment-peptides	22 / 22	100.00	22 / 22	100.00
Polysacharides	8 / 8	100.00	8 / 8	100.00
Total	265 / 265	100.00	259 / 265	97.74

Table S3. Minimum RMSD per system; 95% CIs from Student's t (mean) and χ^2 (SD). Overall: all 265 systems pooled.

Dataset	*N*	Mean	95% CI (mean)	SD	95% CI (SD)
4 Fragments	129	3.0597	2.9347–3.1847	0.7175	0.6393–0.8176
5 Fragments	62	3.4248	3.2987–3.5509	0.4965	0.4219–0.6034
6 Fragments	44	3.8695	3.7438–3.9953	0.4135	0.3416–0.5239
7 Fragments	22	3.8476	3.7083–3.9870	0.3143	0.2418–0.4492
Carbohydrates	8	2.9156	2.4499–3.3812	0.5570	0.3683–1.1336
Overall	265	3.3407	3.2583–3.4231	0.6813	0.6279–0.7449

Table S4. Mean RMSD per system; 95% CIs from Student's t (mean) and χ^2 (SD). Overall: all 265 systems pooled.

Dataset	*N*	Mean	95% CI (mean)	SD	95% CI (SD)
4 Fragments	129	3.4916	3.3809–3.6024	0.6355	0.5663–0.7242
5 Fragments	62	3.8208	3.6930–3.9486	0.5033	0.4277–0.6117
6 Fragments	44	4.2301	4.1125–4.3476	0.3865	0.3194–0.4898
7 Fragments	22	4.2767	4.1766–4.3767	0.2256	0.1735–0.3223
Carbohydrates	8	4.1132	3.4596–4.7669	0.7819	0.5169–1.5913
Overall	265	3.7752	3.6989–3.8515	0.6306	0.5811–0.6894

Table S5. Success rates at selected RMSD thresholds (minimum rmsd_md per system).

Subset	N	≤ 2.0 Å	≤ 2.5 Å	≤ 4.0 Å	≤ 5.0 Å
4 Frag	129	8 (6.20%)	26 (20.16%)	116 (89.92%)	129 (100.0%)
5 Frag	62	0 (0.00%)	2 (3.23%)	55 (88.71%)	62 (100.0%)
6 Frag	44	0 (0.00%)	0 (0.00%)	28 (63.64%)	44 (100.0%)
7 Frag	22	0 (0.00%)	0 (0.00%)	13 (59.09%)	22 (100.0%)
Total	257	8 (3.11%)	28 (10.89%)	212 (82.49%)	257 (100.0%)

Table S6. Typical causes of unsuccessful assemblies during benchmark execution. check_target denotes cases rejected because of inconsistent residue numbering, typically caused by HETATM records disrupting the residue sequence. 2nd chain corresponds to structures in which non-consecutive chain definitions prevent successful topology generation by GROMACS. Incomplete atoms indicate missing or inconsistent atomic definitions in the receptor structure. acpype/ligand refers to failures during topology generation or parameterization of the assembled ligand. Other includes remaining topology-generation issues such as unsupported residue types, force-field incompatibilities, duplicated atoms, or other structural inconsistencies.

Fragments	Assembly rate (%)	check_target	2nd chain	Incomplete atoms	acpype/ligand	Other
4	7.73	16.3%	53.0 %	16.9%	12.2%	1.6%
5	7.54	2.7%	97.3 %	0.0%	0.0%	0.0%
6	9.74	21.5%	30.6 %	23.7%	16.7%	7.5%
7	4.94	44.3%	23.4 %	20.8%	8.3%	3.1%

Table S7. Approximate execution-time comparison (min) between STELLAR, DLPepDock, HPEPDOCK, and DockThor for 10–12-residue peptides.

Nº Residues	**STELLAR**	HPepDock	DockThor	DLPepDock
10	80-120	30-50	Not reported	53
11	80-120	35-55	Not reported	127
12	80-120	40-60	Not reported	117

Table S8. Benchmark comparison between STELLAR and the three competitors' software DLPepDock, HPepDock and DockThor.

PDB ID	Nº Residues	**STELLAR**		HPepDock	DockThor	DLPepDock
		Minimum	Average			
1H6W	10	0.9	1.5	3.6	19.7	1.7
1NTV	10	3.5	3.8	15.7	1.3	6.5
3BRL	10	2.9	3.9	12.5	7.3	12.5
4DS1	10	4.2	4.5	6.3	7.3	7.3
2XFX	11	2.4	3.8	7.0	12.9	6.2
3BFW	11	2.0	2.6	2.0	16.0	1.0
2B9H	12	3.3	3.8	2.1	14.2	9.2
2W10	12	4.5	4.9	15.4	1.7	17.7
